# One-Step Biomimetic Synthesis of the Alkaloids Karachine, Valachine, and Sinometumine E

**DOI:** 10.1021/acs.orglett.5c04172

**Published:** 2025-11-13

**Authors:** Alexander A. Fadeev, Amálie Vanoušková, Martin Kotora

**Affiliations:** Department of Organic Chemistry, Faculty of Science, Charles University, Hlavova 8, 128 00 Praha 2, Czech Republic

## Abstract

An efficient one-step synthesis of the protoberberine alkaloids karachine, valachine, and sinometumine E is described. This transformation relies on the biosynthetic hypothesis featuring vinylogous aldol, Michael, and Mannich addition reactions working in tandem under mild non-enzymatic conditions. The method employs renewable substrates in an atom-economical and operationally simple manner, allowing for a multigram-scale synthesis. The structure of karachine was confirmed crystallographically and showed the intramolecular n→π* interaction that enabled stereoselective nucleophilic addition to the carbonyl group.

## Graphical abstract

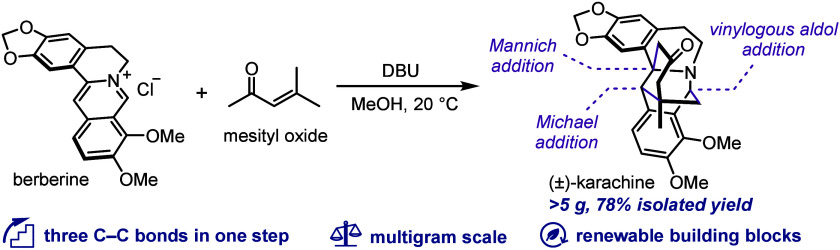

Tetrahydroisoquinolines represent a structurally diverse alkaloid family with many bioactive members and uses in medicine.^1–3^ These alkaloids are constantly in the focus of the synthetic community, which aims to bridge the gap between their preparation and application.^4,5^ This is generally achieved through minimizing synthetic complexity^6–8^ and the use of protecting groups,^9–12^ increasing scalability,^13^ and using renewable feedstocks.^14^ The synthetic challenge naturally increases with the structural complexity of the target molecule, and deciphering the biosynthesis of natural products is a desirable task that often provides the most efficient synthetic solutions.^15–22^

Among tetrahydroisoquinoline alkaloids, karachine (**1**) (Scheme 1) was the first known member with an intricately bridged 6/6/6/6/6/6 protoberberine skeleton found in *Berberis aristata* and named after Karachi, Pakistan.^23^ Karachine and the alkaloids valachine (**2**)^24,25^ and the more recent sinometumine E (**3**)^26,27^ remain among the most structurally complex protoberberines known to date. Although karachine was originally suggested to arise from berberine (**4**) and two acetone molecules,^23^ there is still no evidence of how karachine’s polycyclic scaffold is assembled in nature. Indeed, the scarce content of karachine, valachine, and sinometumine E in natural sources obstructs examination of the origin and properties of these compounds. Thus, sinometumine E only recently showed promising angiogenetic activity through regulating the HIF-1/VEGF pathway *in vivo* and *in vitro*.^27^ Otherwise, the karachine alkaloid family is much less studied compared to its assumed predecessors—the abundant alkaloids berberine and palmatine.^2,3,28^ In this regard, developing the chemical synthesis of karachine and its congeners could shed light on their biosynthesis and provide them in sufficient quantities for further studies.

**Scheme 1:**
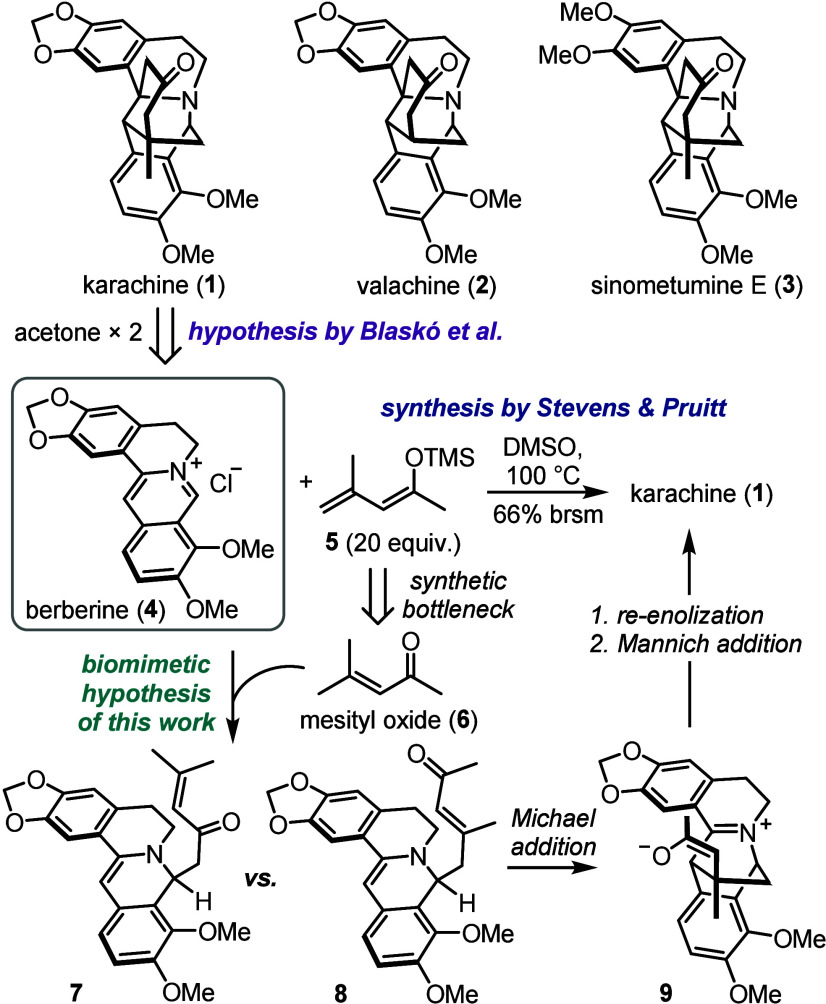
Synthesis and Suggested Biosynthesis of Karachine and Analogs

In 1983, Stevens and Pruitt reported a two-step synthesis of karachine through condensation of berberine with siloxy diene **5** as the key step.^29^ However, this transformation suffers from poor atom economy and results in incomplete conversion of berberine even with a 20-fold excess of labile diene **5**, which is in turn prepared by silylation of mesityl oxide (**6**).^30,31^ This obstructs the preparative applicability and scalability of this approach, and efforts to develop a more efficient synthesis of karachine and related alkaloids are ongoing.^32,33^ Nonetheless, neither synthetic nor biosynthetic formation of karachine directly from berberine and mesityl oxide has been considered, despite the long-known occurrence of both substrates in plants^2,3,34,35^ and their availability from renewable sources.^36^

Mechanistically, this dearomative transformation suggests (a) regioselective vinylogous aldol addition of **6** to **4** with a preference for adduct **8** over **7**, (b) ensuing stereoselective Michael addition in **8** leading to zwitterion **9**, and (c) re-enolization of **9** and subsequent intramolecular Mannich addition to give karachine.^29,37^ Although the synthesis of karachine from **4** and **5** shows that the intramolecular reaction steps are unlikely to be the limiting factors, the desirable one-step synthesis from **4** and **6** requires pairing these steps with the selective activation of an allylic methyl group in **6** under the same conditions. Considering that all known karachine-type alkaloids were isolated in racemic form, we presumed that achieving this activation through selective non-enzymatic enolization could enable a straightforward preparation of karachine, valachine, and sinometumine E from readily available starting materials.

In principle, enolization of mesityl oxide produces both kinetic and thermodynamic dienolates, the ratio of which depends on the reaction conditions.^30,31^ Aiming for the thermodynamic enolization of **6** to achieve the desired vinylogous aldol reaction with **4**, we performed a series of experiments with these compounds in the presence of organic and inorganic bases at room temperature and upon heating (Tables 1 and S1–S3). Thus, heating **4** with **6** in MeCN in the presence of NaOH or KOH gave complex product mixtures containing unstable addition products **7** and **8** in low yields. No reaction was observed with Et_3_N as a weak organic base. The stronger organic base DBU showed a similar result to the alkalis; however, the reaction commenced already at room temperature in an improved yield (20% yield of **7** and 33% yield of **8**). Interestingly, the desired adduct **8** was preferred over **7** in less polar solvents, such as DCM and THF, although the yield of **8** did not exceed 33%.

**Table 1:**
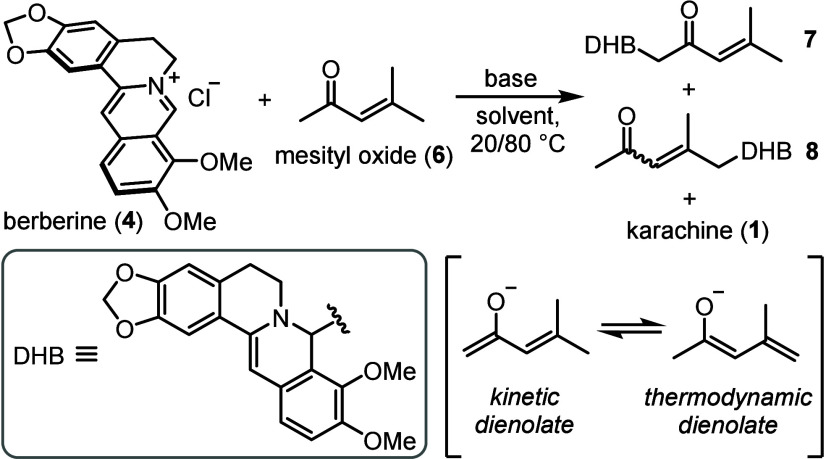
Selected Screening Experiments for the Base-Mediated Reaction of Berberine (**4**) with Mesityl Oxide (**6**)­^a^

			yields (%)		
entry	base	solvent	**7**	**8** ^b^	**1**
1​^c^	NaOH	MeCN	10	19	0
2​^c^	KOH	MeCN	7	12	0
3​^c^	Et_3_N	MeCN	ND^d^	ND^d^	ND^d^
4​^c^	DBU	MeCN	12	18	0
5​	DBU	MeCN	20	33	0
6​	DBU	DCM	9	33	0
7​	DBU	THF	<5	15	0
8​	DBU	AcOH	ND^d^	ND^d^	ND^d^
9​	DBU	EtOH	0	0	70
10​	DBU	^*t*^BuOH	<5	20	0
**11**	**DBU**	**MeOH**	**0**	**0**	**86** ^e^ **(85)**

a The reactions were performed with 0.1 mmol of **4**, 0.2 mmol of **6**, and 0.2 mmol of a base at 20 °C, unless otherwise noted. ^1^H NMR yields are given.

b Combined yields of the stereoisomers (formed in nearly equal amounts).

c At 80 °C.

d Not detected (no reaction was observed).

e The same result was obtained with reduced loadings of DBU (0.15 mmol) and **6** (0.175 mmol); the isolated yield on 0.5 mmol scale is given in parentheses.

Although the instability of compounds **7** and **8** did not allow them to be isolated in the pure state, the attempted chromatography of their crude mixture on silica gel afforded karachine in 4% yield (not detected in the reaction mixture), indicating that little energy is required to convert **8** to **1**. Hoping to incorporate this supposedly acid-catalyzed conversion into the reaction between **4** and **6**, we decided to rely on the acidity of the reaction medium. No reaction was observed in AcOH, evidently due to neutralization of DBU. However, when the reaction was performed in EtOH, neither **7** nor **8** was detected, and karachine was the major reaction product, obtained in 70% yield. At the same time, karachine was not formed in ^*t*^BuOH, suggesting that not only the acidity but also the nucleophilicity of the alcohol are important for the reaction success.^38^ Finally, the yield of karachine was improved to 86% with MeOH as the solvent, and lowering the amount of **6** to 1.75 equiv and the amount of DBU to 1.5 equiv did not affect the reaction’s performance. Presumably, the reversibility of the vinylogous aldol and Michael addition steps in MeOH enables thermodynamic control over the reaction, making karachine the only observed product.

With the optimal reaction conditions in hand, we scaled up the synthesis of karachine and applied the method to the synthesis of valachine and sinometumine E. Luckily, the reaction performed similarly on a 15 mmol scale, providing 5.08 g (78% yield) of karachine (Scheme 2). Under the same reaction conditions, berberine reacted with 3-penten-2-one (**10**) to afford valachine (4.71 g, 75% yield). Likewise, 6.08 g (90% yield) of sinometumine E was prepared starting from palmatine (**11**) and mesityl oxide. All of the prepared alkaloids were spectrally identical to the natural ones (Tables S4–S6).

**Scheme 2:**
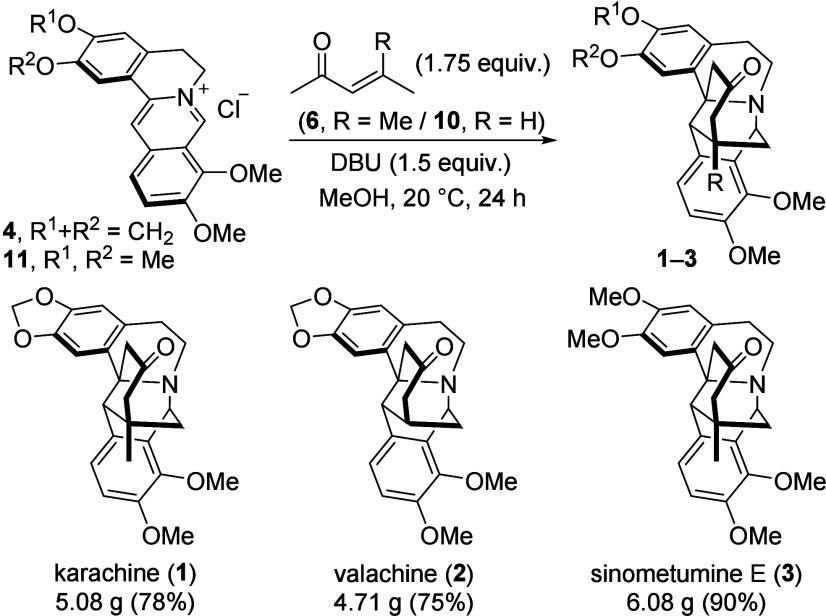
Gram-Scale Syntheses of Karachine, Valachine, and Sinometumine E

Next, we confirmed the structure of karachine unequivocally by single-crystal X-ray diffraction. In the crystal form, the molecule is considerably bent and adopts a saddle shape in which the cyclohexanone ring favors the chair conformation while the piperidine ring adapts the boat conformation (Figure 1). Interestingly, the 2.8 Å distance between the nitrogen atom and the carbon of the C=O group together with the Bürgi–Dunitz angle of 113° reveal the intramolecular n→π* interaction.^39^

**Figure 1:**
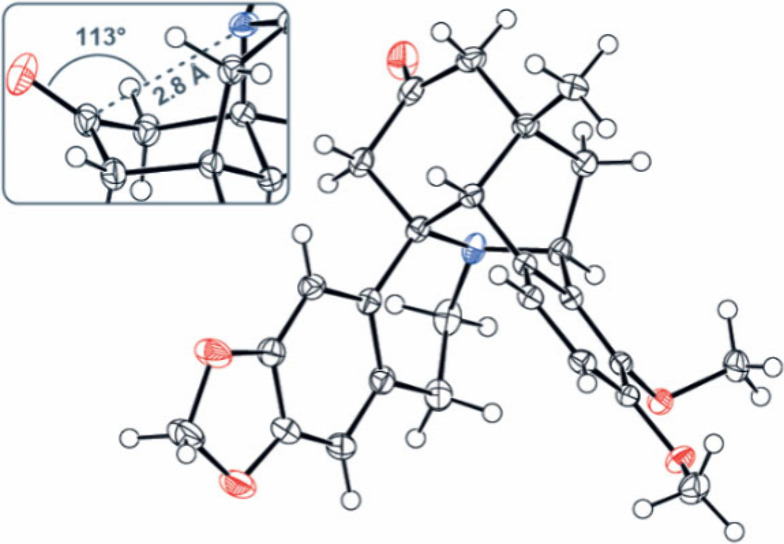
ORTEP representation of karachine (**1**) (50% ellipsoid probability).

The observation of the n→π* donation prompted us to investigate the stereochemistry of the nucleophilic addition to the carbonyl group of karachine. On the one hand, substituted cyclohexanones typically prefer to react axially with small nucleophiles and equatorially with large nucleophiles. On the other hand, the n→π* interaction in karachine could cause small nucleophiles to react equatorially by hindering the axial approach to the cyclohexanone ring, as previously suggested for the borohydride reduction of karachine into dihydrokarachine (**12**).^23^ To ascertain the stereochemical outcome of this transformation, we treated karachine with sodium borohydride to obtain **12** (96% yield) and investigated its crystal structure. The hydroxyl group in **12** was found in the axial position, permitting the 1.9 Å hydrogen bond to the nitrogen atom (Figure 2), thus supporting the previously proposed stereochemistry. Similarly, addition of methylmagnesium bromide to **1** occurred with complete stereocontrol, providing alcohol **13** in 88% yield.

**Figure 2:**
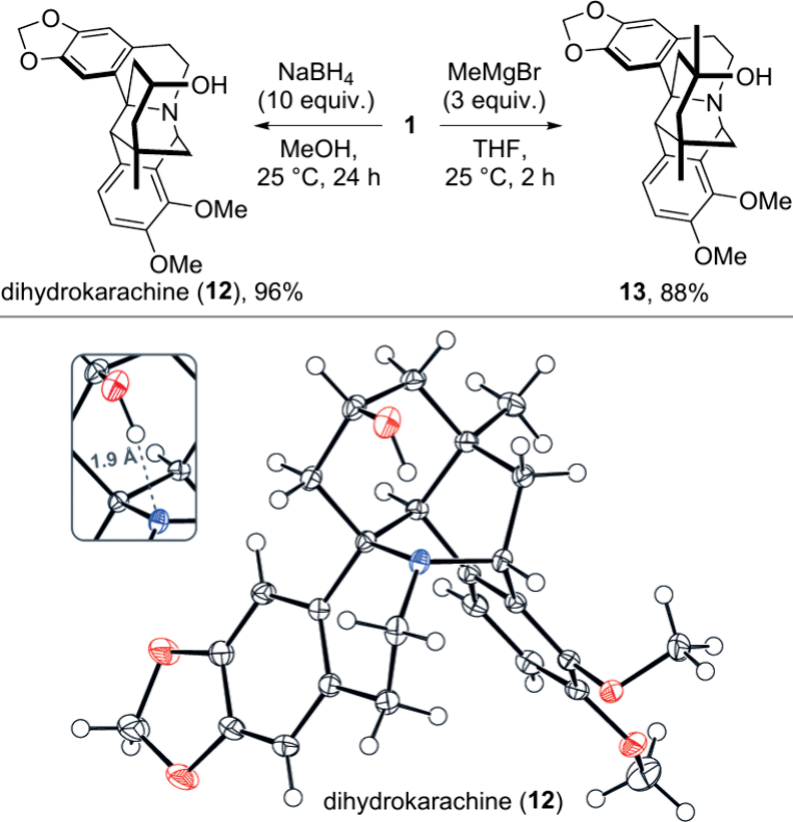
Stereoselective nucleophilic addition to **1** and ORTEP representation of product **12** (50% ellipsoid probability).

In summary, we showed that selective enolization of mesityl oxide using DBU in methanol initiates three consecutive addition reactions to the pyridine ring of berberine that ultimately lead to karachine in a high yield. This straightforward and atom-economical approach is operationally simple, scalable, and applicable to the synthesis of valachine and sinometumine E. By relying on renewable and naturally occurring building blocks, this synthetic route aims to mimic the yet unexplored biosynthesis of these alkaloids. The disclosed crystallographic properties of karachine and its dihydro derivative could help to develop new bioactive substances based on the bridged 6/6/6/6/6/6 protoberberine skeleton.

## Supplementary Material



## Data Availability

The data underlying this study are available in the published article and its Supporting Information.

## Notes

Experimental procedures, compound characterization data, and crystallographic properties of compounds **1** and **12** (PDF)
