# Supplementary material for: One-Step Biomimetic Synthesis of the Alkaloids Karachine, Valachine, and Sinometumine E
Source: Org Lett. 2025 Nov 13;27(47):13040–4. doi: 10.1021/acs.orglett.5c04172 (PMC12670697; doi:10.1021/acs.orglett.5c04172)
Supplement: Supplementary file 1 [file ol5c04172_si_001.pdf]

Supplementary information

for

One-step Biomimetic Synthesis of Alkaloids Karachine, Valachine, and Sinometumine E

Alexander A. Fadeev, Amálie Vanoušková, and Martin Kotora

Table of Contents

1. General 2
2. Experimental procedures and compound characterization 3

2.1. Screening of the reaction between berberine and mesityl oxide 3

2.2. Preparative synthesis of karachine, valachine and sinometumine E 7

3. Comparison of the NMR data between the natural and the synthesized compounds 13
4. Crystallographic data 18
5. Copies of NMR spectra 20
6. References 49

1. General

All reagents and solvents were purchased form commercial sources (Merck, Fluorochem, TCI Chemicals, BLDPharm, Lach-Ner). Berberine chloride hydrate was obtained from Merck and Fluorochem (no difference in quality was observed). Palmatine chloride hydrate was obtained from BLDPharm. Mesityl oxide was obtained from TCI Chemicals (>95% purity; technical grade product showed inferior results). 3-Penten-2-one was obtained from BLDPharm. 1,8-Diazabicyclo[5.4.0]undec-7-ene (DBU) was obtained from Merck and Fluorochem (no difference in quality was observed). Solvents were purified and dried by distillation: tetrahydrofuran (THF) was distilled over sodium in the presence of benzophenone, methanol was purified by regular distillation. Other solvents and all reagents were used without further purification unless otherwise noted. The room temperature was maintained between 20 and 25 °C. Column chromatography was performed on silica gel SiliaFlash® P60 (40−63 μm) from Silicycle. Thin layer chromatography (TLC) was performed on Merck silica gel 60 F254 coated aluminum sheets (254 nm UV light and neutral aqueous $KMnO_4$ were used for visualization). The ^{1}H NMR and ^{13}C NMR spectra were recorded on Bruker AVANCE III HD Spectrometer and Bruker AVANCE NEO 400 Spectrometer (^{1}H at 400 MHz and ^{13}C at 101 MHz) as solutions in $CDCl_3$ and CD_3OD. Chemical shifts are given in parts per million (ppm) and referenced to the residual solvent peaks: $CDCl_3$ (^{1}H, $\delta = 7.26$; ^{13}C, $\delta = 77.2$), CD_3OD (^{1}H, $\delta = 3.31$; ^{13}C, $\delta = 49.0$). Coupling constants *J* are given in Hz (Hertz), splitting patterns are abbreviated as s (singlet), br. s (broad singlet), d (doublet), m (multiplet), dd (doublet of doublets), dt (doublet of triplets), and ddd (doublet of doublets of doublets). Structural assignments were made with additional information from gCOSY, gHSQC, and gHMBC experiments. The NMR spectra of the natural compounds used for the comparison with the synthesized products were simulated in MestreNova software from the previously reported data. The IR spectra were recorded on a Thermo Nicolet Avatar 370 FT-IR spectrometer in KBr powder and are reported in wave numbers (cm^{-1}). The HRMS spectra were measured using Agilent 6530 Accurate-Mass Q-TOF LC/MS, Bruker amaZon SL and Bruker Esquire 3000. Crystallographic data were collected on Bruker D8 VENTURE Kappa Duo PHOTONIII by IμS micro-focus sealed tube. All melting points were determined on a meting point apparatus Kofler KB T300.

2. Experimental procedures and compound characterization

2.1. Screening of the reaction between berberine and mesityl oxide

General screening reaction setup

To a stirred suspension of berberine chloride hydrate (0.1 mmol, 39 mg) in the given solvent (1 mL) was added mesityl oxide (0.2 mmol, 23 μL) followed by the respective base (0.2 mmol). The reaction was stopped after full conversion of berberine was observed (TLC control) or after 24 h regardless of the conversion. In the cases where no product spots were detected by TLC, the mixtures were not analyzed further. In the cases where a product formation was detected, the reaction mixtures were brought to room temperature, filtered from the solid material (if any), and the solid was washed with EtOAc (10 mL). Next, the filtrate was concentrated under reduced pressure at 40−50 °C (mixtures containing high-boiling liquids, such as DMF and DMSO, were additionally high-vacuum dried) and the residue was dissolved in $CDCl_3$ and analyzed by ^{1}H NMR with dibromomethane (0.1 mmol, 7 μL) as the internal standard. The results are shown in Tables S1−S3.

Analysis of the reaction mixture contents

The contents of the reaction mixtures were determined based on the characteristic peaks of the products in the ^{1}H NMR spectra recorded in $CDCl_3$. Karachine (**1**) was identified by the comparison with the spectral values reported for the sample isolated from *Berberis aristata*.[1] The structure of adduct **7** was determined through the comparison with the spectral values of the berberine acetone adduct as a structurally similar reference compound (Figure S1).[2] The stereoisomeric adduct **8** was assigned based on the characteristic chemical shifts and splitting patterns with the aid of homonuclear correlation spectroscopy (^{1}H–^{1}H COSY, Figure S2).

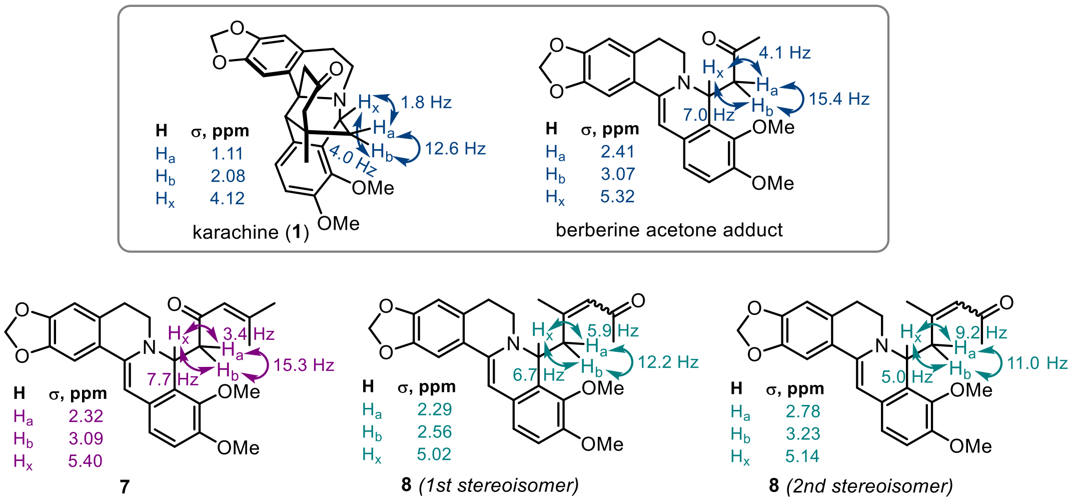

Figure S1. Assignment of the reaction mixture contents by ^{1}H NMR (400 MHz, $CDCl_3$).

Attempted isolation of compounds 7 and 8

1) By chromatographic separation. The reaction of berberine (**4**) with mesityl oxide (**6**) was performed on 0.5 mmol scale under the conditions given in Table S1, Entry 13. Next, volatiles were removed under reduced pressure at 40 °C. Column chromatography of the residue on silica gel (gradient elution from 100% hexanes to 100% EtOAc) only afforded 9 mg (4%) of karachine (**1**) as a light yellow solid (eluted with 6/1 hexanes/EtOAc).

2) By crystallization. Following the procedure for the synthesis of berberine acetone adduct,[2] berberine chloride hydrate (0.5 mmol, 195 mg) was added to a stirred solution of NaOH (5 mmol, 200 mg) in water (1 mL) at room temperature (20 °C). Mesityl oxide (2.2 mmol, 250 μL) was introduced to the formed suspension and the reaction mixture was stirred for 0.5 h. After that, the resulting suspension was vacuum-filtered, and the sticky solid material was washed with 80% aqueous MeOH (30 mL) and discarded (^{1}H NMR analysis showed a complex product mixture). The filtrate was cooled to 5 °C, and the formed precipitate was filtered and washed with 80% aqueous MeOH (5 mL), hexanes (10 mL), and dried *in vacuo* at room temperature to give a pale yellow solid (32 mg). Although further purification of this material by crystallization was not successful, this crude product mainly contained adducts **7** and **8**, the characteristic ^{1}H NMR peaks of which were sufficiently resolved to serve as the reference (Figure S2).

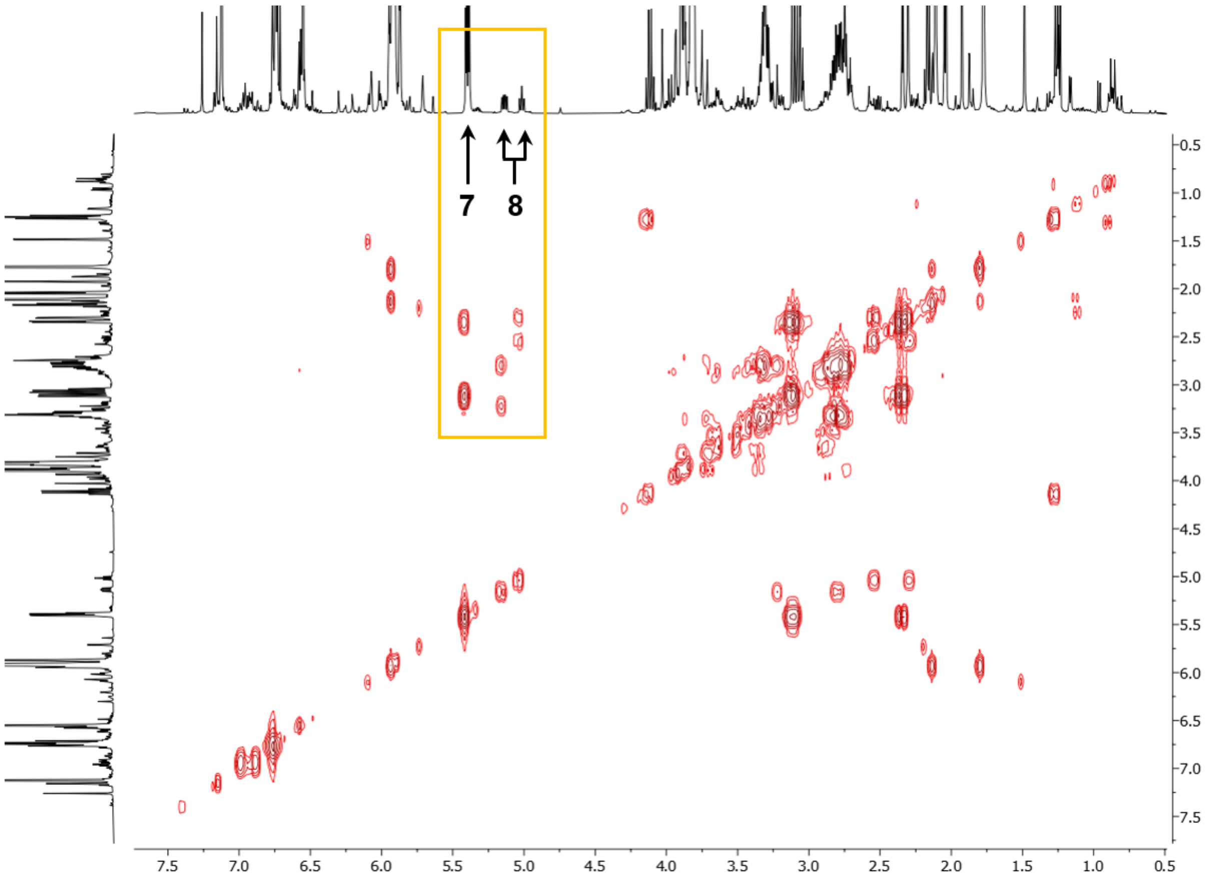

Figure S2. ^{1}H–^{1}H COSY spectrum of the mixture containing **7** and **8**.

Table S1. Screening of organic and inorganic bases (DIPEA = *N*,*N*-diisopropylethylamine, DBU = 1,8-diazabicyclo[5.4.0]undec-7-ene, DABCO = 1,4-diazabicyclo[2.2.2]octane, HMDS = hexamethyldisilazane). The formation of **1** was not observed in these experiments.

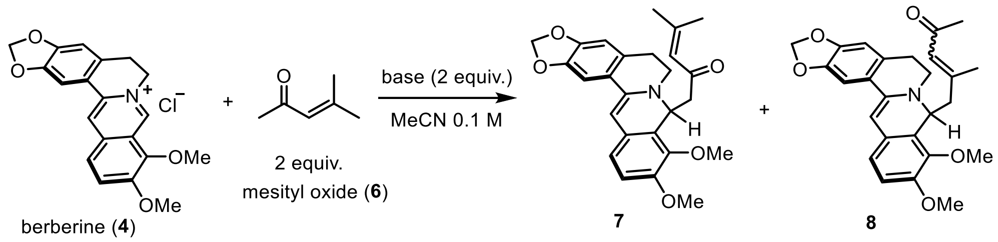

Entry	Base	T (°C)	t (h)	**7**, yield (%)	**8**, yield (%)
1	–	20	24	ND	ND
2	–	80	24	ND	ND
3	Et_3N	20	24	ND	ND
4	Et_3N	80	24	ND	ND
5	$^{i}Pr_2NH$	20	24	ND	ND
6	$^{i}Pr_2NH$	80	24	ND	ND
**7**	DIPEA	20	24	ND	ND
8	DIPEA	80	24	ND	ND
9	morpholine	20	24	ND	ND
10	morpholine	80	24	ND	ND
11	pyridine	20	24	ND	ND
12	pyridine	80	24	ND	ND
13	DBU	20	2	20	33
14	DBU	80	1	12	18
15	DABCO	20	24	ND	ND
16	DABCO	80	24	ND	ND
17	HMDS	20	24	ND	ND
18	HMDS	80	24	ND	ND
19	NaOH	20	24	ND	ND
20	NaOH	80	3	10	19
21	KOH	20	24	ND	ND
22	KOH	80	3	7	12
23	K_2CO_3	20	24	ND	ND
24	K_2CO_3	80	24	<5	<5
25	Cs_2CO_3	20	24	ND	ND
26	Cs_2CO_3	80	24	8	12

ND – not detected.

Table S2. Screening of solvents (THF = tetrahydrofuran, DCM = dichloromethane, DMF = *N*,*N*-dimethylformamide, DMSO = dimethyl sulfoxide).

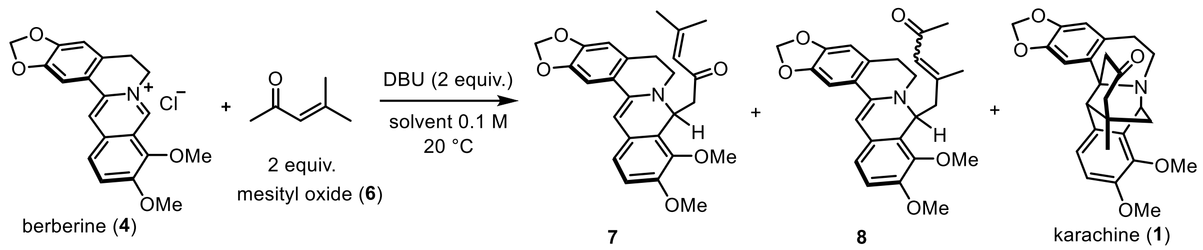

Entry	Solvent	T (°C)	**7**, yield (%)	**8**, yield (%)	**1**, yield (%)
1	toluene	24	ND	ND	ND
2	THF	24	<5	15	0
3	dioxane	24	<5	32	0
4	DCM	24	9	33	0
5	EtOH	24	0	0	70
6	DMF[a]	1	ND	ND	ND
7	DMSO[a]	1	ND	ND	ND
8	MeOH	2	0	0	43
9	MeOH	24	0	0	86
10	^{t}BuOH	24	<5	20	0
11	AcOH	24	ND	ND	ND

[a] increasingly complex product mixtures were formed in these solvents.

Table S3. Screening of the amounts of the reagents and additional base screening.

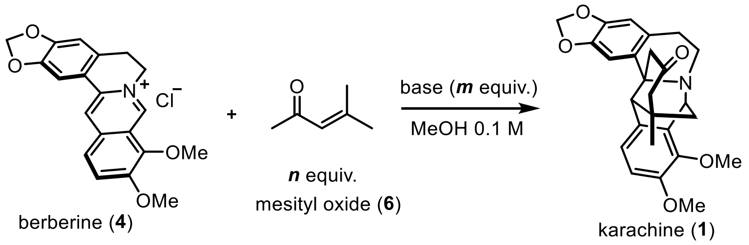

Entry	Base	*n*	*m*	t (h)	**1**, yield (%)
1	DBU	2	1.5	24	86
2	DBU	2	1.25	24	77
3	DBU	2	1.1	24	70
4	DBU	1.75	1.5	24	86 (85)[a]
5	DBU	1.5	1.5	24	83
6	DBU	1.25	1.5	24	71
7	DBU	1.1	1.5	24	64
8	NaOH	1.75	1.5	24	63
9	K_2CO_3	1.75	1.5	24	56
10	Cs_2CO_3	1.75	1.5	24	48

[a] isolated yield on 0.5 mmol scale.

2.2. Preparative synthesis of karachine, valachine and sinometumine E

9,10-Dimethoxy-13-methyl-5,6,8,12b,13,14-hexahydro-8,13-methano[1,3]dioxolo-[4',5':6,7]isoquinolino[1,2-*e*]phenanthridin-15(16*H*)-one (karachine, 1)

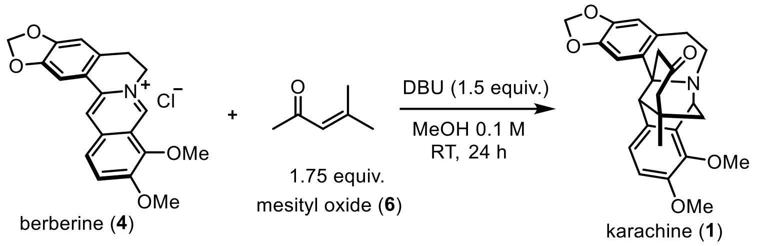

Small-scale synthesis: To a stirred suspension of berberine chloride hydrate (0.5 mmol, 195 mg) in MeOH (5 mL) was added mesityl oxide (0.875 mmol, 1.75 equiv., 86 mg, 100 μL) followed by a dropwise addition of DBU (0.75 mmol, 1.5 equiv., 114 mg, 112 μL). The reaction mixture was stirred for 24 h at room temperature (20 °C). Next, volatiles were removed under reduced pressure at 40–50 °C. Column chromatography of the residue on silica gel (6/1 hexanes/EtOAc) gave 184 mg (85%) of karachine as a light yellow solid.

Multigram-scale synthesis (Figure S3): In a 500 mL round bottom flask, berberine chloride hydrate (15 mmol, 5.85 g) was suspended in MeOH (150 mL). Mesityl oxide (26.25 mmol, 1.75 equiv., 2.57 g, 3.0 mL) was added in a single portion, and the flask was placed in a room temperature water bath (20 °C). Next, DBU (22.5 mmol, 1.5 equiv., 3.42 g, 3.4 mL) was added dropwise in the course of 5 min (exothermic). The flask was stoppered, and the formed dark red solution was stirred for 24 h at room temperature (20 °C). After that, the mixture was cooled to 5 °C, filtered, washed with cold MeOH (30 mL) and air-dried to give karachine as a light yellow solid (3.92 g, 60%). The filtrate was concentrated under reduced pressure, and the residue was dissolved in DCM (50 mL). This solution was then filtered through a pad of silica gel (50 g, d = 40 mm, h = 100 mm, pre-wetted with hexanes) and washed with 1:1 hexanes/EtOAc mixture (300 mL). The clear pale yellow solution was then concentrated under reduced pressure to give additional 1.74 g of the product (27%). The combined solid material was recrystallized from EtOAc to give 5.08 g (78%) of karachine as light yellow crystals (leaflets).

TLC R_f = 0.43 (3/2 hexanes/EtOAc);

M.p. 146–147 °C, [lit.[1] 146–148 °C];

^{1}H NMR (400 MHz, $CDCl_3$): δ = 6.72 (s, 1H), 6.55 (d, *J* = 8.0 Hz, 1H), 6.50 (d, *J* = 8.0 Hz, 1H), 6.15 (s, 1H), 5.85 (d, *J* = 1.5 Hz, 1H), 5.80 (d, *J* = 1.5 Hz, 1H), 4.05 (dd, *J* = 4.0, 1.9 Hz, 1H), 3.81 (s, 3H), 3.75 (s, 3H), 3.08 (ddd, *J* = 13.4, 10.8, 3.6 Hz, 1H), 3.06 (s, 1H), 2.97 (dt, *J* = 13.4, 4.3 Hz, 1H), 2.72 (d, *J* = 14.0 Hz, 1H), 2.68 (d, *J* = 14.0 Hz, 1H), 2.49 (d, *J* = 14.3 Hz, 1H), 2.44 (d, *J* = 14.3 Hz, 1H), 2.25 (ddd, *J* = 15.5, 10.8, 4.3 Hz, 1H), 2.13 – 2.03 (m, 2H), 1.10 (dd, *J* = 12.5, 1.9 Hz, 1H), 0.80 (s, 3H);

^{1}H NMR (400 MHz, CD_3OD): δ = 6.92 (s, 1H), 6.63 (d, *J* = 8.0 Hz, 1H), 6.59 (d, *J* = 8.0 Hz, 1H), 6.15 (s, 1H), 5.81 (d, *J* = 1.5 Hz, 1H), 5.80 (d, *J* = 1.5 Hz, 1H), 4.05 (dd, *J* = 4.0, 1.9 Hz, 1H), 3.80 (s, 3H), 3.75 (s, 3H), 3.06 (ddd, *J* = 13.4, 10.8, 3.6 Hz, 1H), 2.97 (dt, *J* = 13.4, 4.3

Hz, 1H), 2.92 (d, J = 14.0 Hz, 1H), 2.70 – 2.56 (m, 2H), 2.37 (dd, J = 14.0, 2.4 Hz, 1H), 2.25 (ddd, J = 15.5, 10.8, 4.3 Hz, 1H), 2.06 (dt, J = 15.5, 3.6 Hz, 1H), 2.03 – 1.98 (m, 2H), 1.09 (dd, J = 12.5, 1.9 Hz, 1H), 0.81 (s, 3H);
^{13}C{^{1}H} NMR (101 MHz, $CDCl_3$): δ = 209.3, 151.0, 146.1, 145.1, 142.4, 134.6, 133.5, 131.1, 130.0, 122.5, 109.9, 108.5, 105.1, 100.7, 63.5, 61.2, 55.7, 55.4, 53.9, 53.5, 52.7, 47.3, 43.5, 36.1, 31.6, 26.6;
IR (KBr): ṽ = 2956, 2920, 2858, 1707, 1485, 1296, 1250, 1217, 1086, 1068, 1032, 935, 796 cm^{-1};
HRMS (ESI): m/z calcd for $C_{26}H_{28}NO_5^+$: 434.1962 $[M+H]^+$; found: 434.1968.
The recorded spectroscopic values agree with the previously reported data (see Table S4).[1]

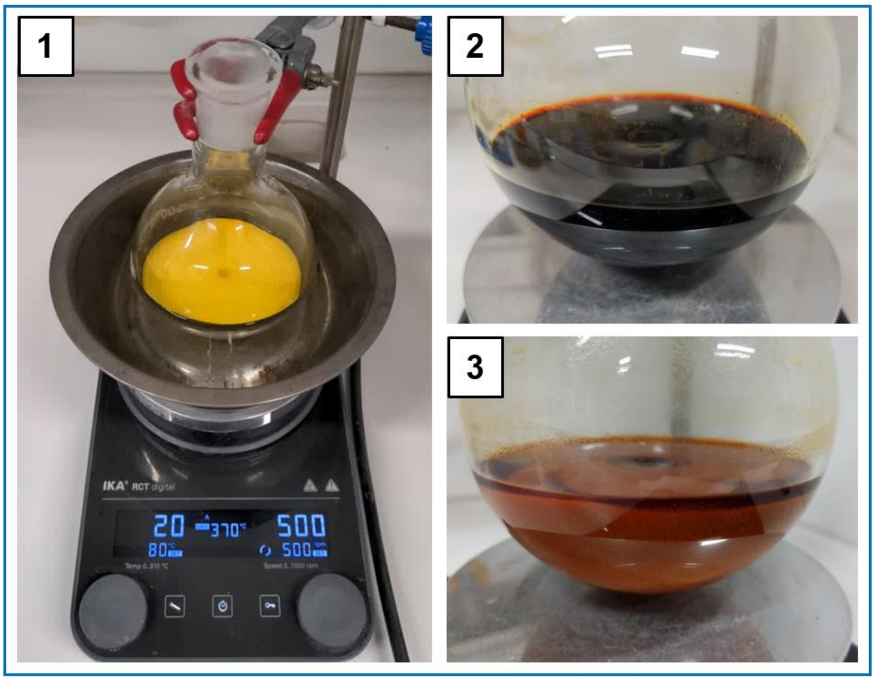

Figure S3. The progress of the multigram-scale synthesis: (1) Berberine and mesityl oxide in MeOH; (2) Berberine dissolves shortly after DBU is added; (3) After 24 hours karachine precipitates.

9,10-Dimethoxy-5,6,8,12b,13,14-hexahydro-8,13-methano[1,3]dioxolo[4',5':6,7]-isoquinolino[1,2-*e*]phenanthridin-15(16*H*)-one (valachine, 2)

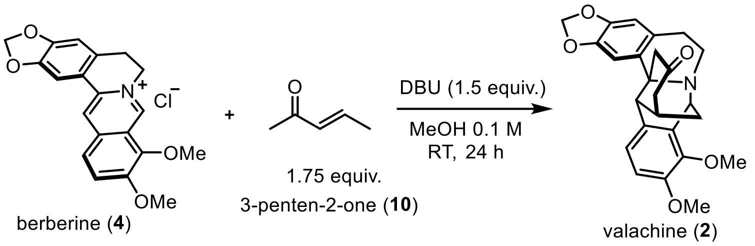

Small-scale synthesis: To a stirred suspension of berberine chloride hydrate (0.5 mmol, 195 mg) in MeOH (5 mL) was added 3-penten-2-one (0.875 mmol, 1.75 equiv., 74 mg, 85 μL) followed by a dropwise addition of DBU (0.75 mmol, 1.5 equiv., 114 mg, 112 μL). The reaction mixture was stirred for 24 h at room temperature (20 °C). Next, volatiles were removed under reduced pressure at 40−50 °C. Column chromatography of the residue on silica gel (5/1 hexanes/EtOAc) gave 123 mg (59%) of valachine as a light yellow solid.

Multigram-scale synthesis: In a 500 mL round bottom flask, berberine chloride hydrate (15 mmol, 5.85 g) was suspended in MeOH (150 mL). After that, 3-penten-2-one (26.25 mmol, 1.75 equiv., 2.21 g, 2.6 mL) was added in a single portion, and the flask was placed in a room temperature water bath (20 °C). Next, DBU (22.5 mmol, 1.5 equiv., 3.42 g, 3.4 mL) was added dropwise in the course of 5 min (exothermic). The flask was stoppered, and the formed dark red solution was stirred for 24 h at room temperature (20 °C). After that, the mixture was cooled to 5 °C, filtered, washed with cold MeOH (30 mL) and air-dried to give valachine as a light yellow solid (4.07 g, 65%). The filtrate was concentrated under reduced pressure, and the residue was purified by column chromatography on silica gel (5/1 hexanes/EtOAc) to afford additional 640 mg (10%) of valachine as a light yellow solid. The combined solid material (4.71 g, 75%) was sufficiently pure without additional purification.

TLC R_f = 0.43 (3/2 hexanes/EtOAc);

M.p. 240−242 °C, [lit.[3] 237−238 °C];

^{1}H NMR (400 MHz, $CDCl_3$): δ = 6.72 (s, 1H), 6.59 (d, *J* = 8.0 Hz, 1H), 6.52 (d, *J* = 8.0 Hz, 1H), 6.16 (s, 1H), 5.85 (d, *J* = 1.5 Hz, 1H), 5.81 (d, *J* = 1.5 Hz, 1H), 4.09 (dd, *J* = 4.0, 2.0 Hz, 1H), 3.82 (s, 3H), 3.75 (s, 3H), 3.41 (d, *J* = 3.3 Hz, 1H), 3.09 (ddd, *J* = 13.5, 10.7, 3.6 Hz, 1H), 2.96 (dt, *J* = 13.5, 4.2 Hz, 1H), 2.79 – 2.64 (m, 3H), 2.57 – 2.50 (m, 1H), 2.40 – 2.32 (m, 1H), 2.26 (ddd, *J* = 15.6, 10.7, 4.2 Hz, 1H), 2.10 (dt, *J* = 15.6, 3.6 Hz, 1H), 1.89 (dt, *J* = 12.7, 3.8 Hz, 1H), 1.63 (ddd, *J* = 12.7, 11.0, 2.0 Hz, 1H);

^{1}H NMR (400 MHz, CD_3OD): δ = 6.92 (s, 1H), 6.64 – 6.60 (m, 2H), 6.16 (s, 1H), 5.81 (d, *J* = 1.5 Hz, 1H), 5.80 (d, *J* = 1.5 Hz, 1H), 4.07 (dd, *J* = 4.0, 2.0 Hz, 1H), 3.80 (s, 3H), 3.74 (s, 3H), 3.62 (d, *J* = 3.3 Hz, 1H), 3.07 (ddd, *J* = 13.5, 10.7, 3.6 Hz, 1H), 3.00 – 2.91 (m, 2H), 2.90 – 2.87 (m, 1H), 2.62 (dd, *J* = 14.1, 2.5 Hz, 1H), 2.43 (dt, *J* = 14.4, 2.5 Hz, 1H), 2.39 – 2.23 (m, 2H), 2.07 (dt, *J* = 15.6, 3.6 Hz, 1H), 1.82 (dt, *J* = 12.7, 3.8 Hz, 1H), 1.61 (ddd, *J* = 12.7, 11.0, 2.0 Hz, 1H);

^{13}C{^{1}H} NMR (101 MHz, $CDCl_3$): δ = 209.7, 151.0, 146.1, 145.2, 142.5, 135.5, 133.5, 132.3, 130.1, 121.0, 110.0, 108.5, 105.2, 100.7, 64.0, 61.2, 55.7, 54.0, 52.1, 47.8, 47.6, 46.6, 36.8, 31.9, 26.7;
IR (KBr): ṽ = 2947, 2902, 2814, 1703, 1483, 1257, 1221, 1092, 1070, 1026, 985, 928, 818 cm^{-1};
HRMS (ESI): m/z calcd for $C_{25}H_{26}NO_5^+$: 420.1805 $[M+H]^+$; found: 420.1803.
The recorded spectroscopic values agree with the previously reported data (see Table S5).[3,4]

2,3,9,10-Tetramethoxy-13-methyl-5,6,8,12b,13,14-hexahydro-8,13-methanoisoquinolino[1,2-*e*]phenanthridin-15(16*H*)-one (sinometumine E, 3)

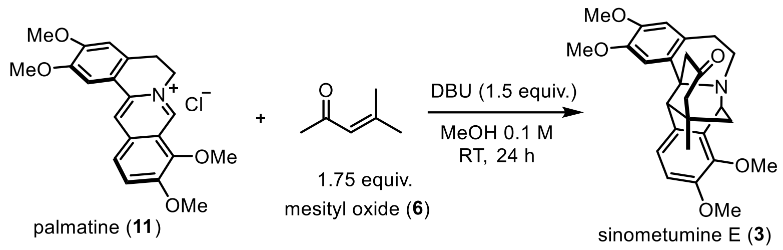

Small-scale synthesis: To a stirred suspension of palmatine chloride hydrate (0.5 mmol, 203 mg) in MeOH (5 mL) was added mesityl oxide (0.875 mmol, 1.75 equiv., 86 mg, 100 μL) followed by a dropwise addition of DBU (0.75 mmol, 1.5 equiv., 114 mg, 112 μL). The reaction mixture was stirred for 24 h at room temperature (20 °C). Next, volatiles were removed under reduced pressure at 40−50 °C. Column chromatography of the residue on silica gel (4/1 hexanes/EtOAc) gave 184 mg (82%) of sinometumine E as a light yellow solid.
Multigram-scale synthesis: In a 500 mL round bottom flask, palmatine chloride hydrate (15 mmol, 6.09 g) was suspended in MeOH (150 mL). Mesityl oxide (26.25 mmol, 1.75 equiv., 2.57 g, 3.0 mL) was added in a single portion, and the flask was placed in a room temperature water bath (20 °C). Next, DBU (22.5 mmol, 1.5 equiv., 3.42 g, 3.4 mL) was added dropwise in the course of 5 min (exothermic). The flask was stoppered, and the formed dark red solution was stirred for 24 h at room temperature (20 °C). After that, volatiles were removed under reduced pressure at 40−50 °C. Column chromatography of the residue on silica gel (4/1 hexanes/EtOAc) gave 6.08 g (90%) of sinometumine E as a light yellow solid.
TLC R_f = 0.28 (2/1 hexanes/EtOAc);
M.p. 112−113 °C;
^{1}H NMR (400 MHz, $CDCl_3$): δ = 6.68 (s, 1H), 6.45 (d, *J* = 8.0 Hz, 1H), 6.42 (d, *J* = 8.0 Hz, 1H), 6.14 (s, 1H), 4.03 (dd, *J* = 3.9, 1.9 Hz, 1H), 3.86 (s, 3H), 3.78 (s, 3H), 3.69 (s, 3H), 3.66 (s, 3H), 3.07 (s, 1H), 3.08 (ddd, *J* = 13.4, 10.9, 3.6 Hz, 1H), 2.99 (dt, *J* = 13.4, 4.3 Hz, 1H), 2.73 (d, *J* = 14.0 Hz, 1H), 2.70 (d, *J* = 14.0 Hz, 1H), 2.48 (d, *J* = 14.3 Hz, 1H), 2.44 (d, *J* = 14.3 Hz, 1H), 2.25 (ddd, *J* = 15.7, 11.0, 4.3 Hz, 1H), 2.10 – 2.01 (m, 2H), 1.08 (dd, *J* = 12.5, 1.9 Hz, 1H), 0.79 (s, 3H);
^{1}H NMR (400 MHz, CD_3OD): δ = 6.95 (s, 1H), 6.56 (d, *J* = 8.0 Hz, 1H), 6.59 (d, *J* = 8.0 Hz, 1H), 6.24 (s, 1H), 4.06 (dd, *J* = 3.9, 1.9 Hz, 1H), 3.85 (s, 3H), 3.80 (s, 3H), 3.72 (s, 3H), 3.64

(s, 3H), 3.37 (s, 1H), 3.08 (ddd, *J* = 13.4, 10.9, 3.6 Hz, 1H), 3.04 – 2.94 (m, 1H), 2.98 (d, *J* = 14.0 Hz, 1H), 2.67 (d, *J* = 14.0 Hz, 1H), 2.62 (dd, *J* = 14.0, 2.4 Hz, 1H), 2.38 (dd, *J* = 14.0, 2.4 Hz, 1H), 2.28 (ddd, *J* = 15.5, 11.0, 4.3 Hz, 1H), 2.07 (dt, *J* = 15.5, 3.4 Hz, 1H), 2.01 (dd, *J* = 12.5, 3.9 Hz, 1H), 1.10 (dd, *J* = 12.5, 1.9 Hz, 1H), 0.81 (s, 3H);
^{13}C{^{1}H} NMR (101 MHz, $CDCl_3$): δ = 209.1, 150.8, 147.2, 146.6, 142.1, 134.4, 132.0, 131.0, 128.8, 122.3, 111.1, 109.7, 108.3, 62.9, 61.0, 56.2, 55.48, 55.46, 55.3, 53.42, 53.36, 52.5, 47.1, 43.5, 35.9, 31.4, 25.8;
^{13}C{^{1}H} NMR (101 MHz, CD_3OD): δ = 213.1, 152.3, 149.0, 148.3, 143.5, 135.8, 133.8, 132.8, 130.1, 123.8, 112.6, 111.2, 110.8, 64.5, 61.5, 56.9, 56.1, 55.9, 54.2, 54.14, 54.13, 53.9, 48.3, 44.9, 37.2, 31.7, 26.8;
IR (KBr): ṽ = 2931, 2856, 2831, 1711, 1514, 1489, 1454, 1255, 1227, 1132, 1082, 1020, 984, 820 cm^{-1};
HRMS (ESI): m/z calcd for $C_{27}H_{32}NO_5^+$: 450.2275 $[M+H]^+$; found: 450.2266.
The recorded spectroscopic values agree with the previously reported data (see Table S6).[5]

9,10-Dimethoxy-13-methyl-5,6,8,12b,13,14,15,16-octahydro-8,13-methano[1,3]dioxolo-[4',5':6,7]isoquinolino[1,2-*e*]phenanthridin-15-ol (dihydrokarachine, 12)

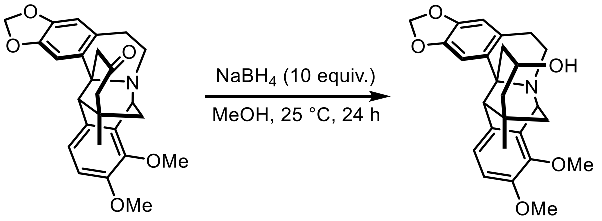

karachine (**1**) dihydrokarachine (**12**), 96%

To a stirred suspension of karachine (0.69 mmol, 300 mg) in MeOH (7 mL) was added sodium borohydride (6.9 mmol, 10 equiv., 262 mg) in portions over 30 minutes. The reaction mixture was stirred for 24 hours at room temperature (25 °C). The reaction was quenched with saturated aqueous NH_4Cl (5 mL) and the product was extracted with DCM (3×10 mL). The combined organic extracts were dried over anhydrous $MgSO_4$ and concentrated under reduced pressure. Column chromatography of the residue on silica gel (2/1 hexanes/EtOAc) gave 289 mg (96%) of the title compound as a colorless solid.
TLC R_f = 0.45 (1/1 hexanes/EtOAc);
M.p. 87−88 °C;
^{1}H NMR (400 MHz, $CDCl_3$): δ = 7.21 (br. s, 1H), 6.61 (s, 1H), 6.51 (d, *J* = 8.2 Hz, 1H), 6.48 (d, *J* = 8.2 Hz, 1H), 6.16 (s, 1H), 5.83 (d, *J* = 1.4 Hz, 1H), 5.79 (d, *J* = 1.4 Hz, 1H), 4.21 (dd, *J* = 4.3, 1.7 Hz, 1H), 4.17 – 4.11 (m, 1H), 3.81 (s, 3H), 3.75 (s, 3H), 3.19 (ddd, *J* = 13.0, 10.5, 3.7 Hz, 1H), 2.97 (dt, *J* = 13.0, 4.2 Hz, 1H), 2.87 (dd, *J* = 12.9, 4.3 Hz, 1H), 2.63 (s, 1H), 2.37 – 2.16 (m, 4H), 1.87 (dd, *J* = 14.4, 4.0 Hz, 1H), 1.66 (dd, *J* = 14.3, 4.0 Hz, 1H), 0.94 (dd, *J* = 12.9, 1.7 Hz, 1H), 0.72 (s, 3H);

$^{13}C\{^1H\}$ NMR (101 MHz, $CDCl_3$): δ = 150.8, 146.1, 144.9, 142.4, 135.1, 133.9, 132.2, 129.5, 122.4, 110.0, 108.2, 105.1, 100.6, 68.6, 61.2, 60.9, 55.7, 54.0, 53.7, 47.7, 45.8, 42.9, 41.8, 32.4, 31.6, 26.5;
IR (KBr): ṽ = 3172, 2951, 2918, 2864, 1504, 1483, 1375, 1261, 1225, 1128, 1022, 984, 939 cm^{-1};
HRMS (ESI): m/z calcd for $C_{26}H_{30}NO_5^+$: 436.2118 $[M+H]^+$; found: 436.2106.
The recorded spectroscopic values agree with the previously reported data.[1]

9,10-Dimethoxy-13,15-dimethyl-5,6,8,12b,13,14,15,16-octahydro-8,13-methano[1,3]dioxolo[4',5':6,7]isoquinolino[1,2-*e*]phenanthridin-15-ol (13)

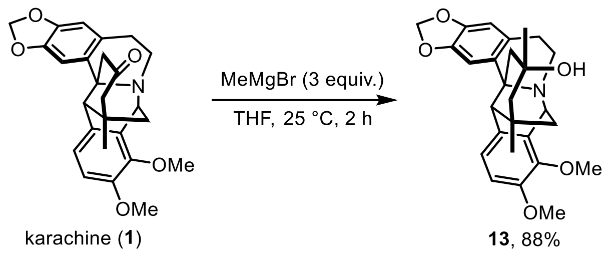

To a stirred solution of karachine (0.69 mmol, 300 mg) in dry THF (7 mL) was added methylmagnesium bromide solution (3.0 M in diethyl ether, 2.08 mmol, 3 equiv., 0.7 mL) dropwise at 0 °C. The reaction mixture was stirred at room temperature for 2 h. The reaction was quenched with saturated aqueous NH_4Cl (5 mL) and the product was extracted with DCM (3×10 mL). The combined organic extracts were dried over anhydrous $MgSO_4$ and concentrated under reduced pressure. Column chromatography of the residue on silica gel (2/1 hexanes/EtOAc) gave 275 mg (88%) of the title compound as a colorless solid.
TLC R_f = 0.44 (1/1 hexanes/EtOAc);
M.p. 92−94 °C;
^1H NMR (400 MHz, $CDCl_3$): δ = 7.74 (br. s, 1H), 6.61 (s, 1H), 6.49 (d, *J* = 8.1 Hz, 1H), 6.46 (d, *J* = 8.1 Hz, 1H), 6.13 (s, 1H), 5.77 (d, *J* = 1.5 Hz, 1H), 5.73 (d, *J* = 1.5 Hz, 1H), 4.20 (dd, *J* = 4.3, 1.4 Hz, 1H), 3.77 (s, 3H), 3.69 (s, 3H), 3.13 (ddd, *J* = 13.0, 10.2, 4.0 Hz, 1H), 2.91 (dt, *J* = 13.0, 4.0 Hz, 1H), 2.86 (dd, *J* = 12.9, 4.3 Hz, 1H), 2.56 (s, 1H), 2.30 (ddd, *J* = 15.7, 10.2, 4.0 Hz, 1H), 2.18 (dt, *J* = 15.7, 4.0 Hz, 1H), 2.01 – 1.90 (m, 2H), 1.68 (d, *J* = 14.1 Hz, 1H), 1.50 (d, *J* = 13.9 Hz, 1H), 1.16 (s, 3H), 0.87 (dd, *J* = 12.9, 1.4 Hz, 1H), 0.68 (s, 3H);
$^{13}C\{^1H\}$ NMR (101 MHz, $CDCl_3$): δ = 150.6, 146.0, 144.8, 142.3, 134.8, 133.7, 131.9, 129.4, 122.3, 109.9, 108.0, 105.0, 100.5, 70.5, 61.3, 61.0, 55.5, 53.5, 53.4, 50.9, 48.3, 47.6, 40.7, 32.4, 32.1, 31.6, 26.5;
IR (KBr): ṽ = 3180, 2956, 2924, 2831, 1483, 1373, 1265, 1221, 1138, 1072, 1036, 984, 935, 916 cm^{-1};
HRMS (ESI): m/z calcd for $C_{27}H_{32}NO_5^+$: 450.2275 $[M+H]^+$; found: 450.2277.

3. Comparison of the NMR data between the natural and the synthesized compounds

Table S4. ^{1}H and ^{13}C NMR characteristics of natural and synthetic karachine. The chemical shift values are given in ppm. Coupling constants in Hz are given in parenthesis.

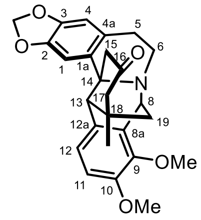

karachine (**1**)

No.	δ_H, nat. ($CDCl_3$)[1]	δ_H, synt. ($CDCl_3$)	δ_C, synt. ($CDCl_3$)
1	6.73, s	6.72, s	105.1
1a	-	-	133.5
2	-	-	146.1
3	-	-	145.1
4	6.17, s	6.15, s	108.5
4a	-	-	130.0
5	2.30 – 2.22, m	2.25, ddd (15.5, 10.8, 4.3); 2.13 – 2.03, m	26.6
6	3.10 – 2.97, m	3.08, ddd (13.4, 10.8, 3.6); 2.97, dt (13.4, 4.3)	47.3
8	4.12, q (4.0, 1.8)	4.05, dd (4.0, 1.9)	52.7
8a	-	-	134.6
9	-	-	142.4
10	-	-	151.0
11	6.52, d (8.2)	6.50, d (8.0)	109.9
12	6.55, d (8.2)	6.55, d (8.0)	122.5
12a	-	-	131.1
13	3.07, s	3.06, s	53.5
14	-	-	63.5
15	2.72, d (14.3); 2.70, d (14.3)	2.72, d (14.0); 2.68, d (14.0)	53.9
16	-	-	209.3
17	2.48, d (14.0); 2.46, d (14.0)	2.49, d (14.3); 2.44, d (14.3)	55.4
18	-	-	36.1
19	2.08, q (12.6, 4.0); 1.11, q (12.6, 1.8)	2.13 – 2.03, m; 1.10, dd (12.5, 1.9)	43.5
2,3-OCH_2O	5.87, d (1.5); 5.82, d (1.5)	5.85, d (1.5); 5.80, d (1.5)	100.7
9-OMe	3.82, s	3.81, s	61.2
10-OMe	3.77, s	3.75, s	55.7
18-Me	0.82, s	0.80, s	31.6

Table S5. ^{1}H and ^{13}C NMR characteristics of natural and synthetic valachine. The chemical shift values are given in ppm. Coupling constants in Hz are given in parenthesis.

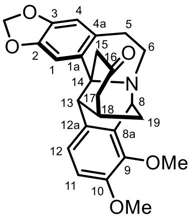

valachine (**2**)

No.	δ_H, nat. (CD_3OD*)[4]	δ_H, synt. ($CDCl_3$)	δ_C, nat. (CD_3OD*)[4]	δ_C, synt. ($CDCl_3$)
1	6.72, s	6.72, s	105.1	105.2
1a	-	-	130.3	133.5
2	-	-	145.0	146.1
3	-	-	145.3	145.2
4	6.18, s	6.16, s	108.6	108.5
4a	-	-	133.6	130.1
5	2.25, m	2.26, ddd (15.6, 10.7, 4.2); 2.10, dt (15.6, 3.6)	26.8	26.7
6	3.09, m; 2.96, m	3.09, ddd (13.5, 10.7, 3.6); 2.96, dt (13.5, 4.2)	47.8	47.6
8	4.10, d	4.09, dd (4.0, 2.0)	52.2	52.1
8a	-	-	135.6	135.5
9	-	-	142.7	142.5
10	-	-	151.1	151.0
11	6.53, d (8.04)	6.52, d (8.0)	110.2	110.0
12	6.58, d (7.9)	6.59, d (8.0)	121.1	121.0
12a	-	-	132.4	132.3
13	3.42, d (3.3)	3.41, d (3.3)	46.7	46.6
14	-	-	64.1	64.0
15	2.73, d (2.3); 2.69, d (2.3)	2.79 – 2.64, m	54.1	54.0
16	-	-	209.7	209.7
17	2.73, d (2.3); 2.55, d (2.3)	2.79 – 2.64, m; 2.57 – 2.50, m	47.7	47.8
18	2.12, m	2.40 – 2.32, m	32.0	31.9
19	2.00, m; 1.66, m	1.89, dt (12.7, 3.8); 1.63, ddd (12.7, 11.0, 2.0)	36.9	36.8
2,3-OCH_2O	5.87, d; 5.82, d	5.85, d (1.5); 5.81, d (1.5)	100.7	100.7
9-OMe	3.82, s	3.82, s	61.2	61.2
10-OMe	3.77, s	3.75, s	58.6	55.7

* CD_3OD was given as the solvent in the original report, yet the spectra appear to be recorded in $CDCl_3$ or a similar solvent

Table S6. ^{1}H and ^{13}C NMR characteristics of natural and synthetic sinometumine E. The chemical shift values are given in ppm. Coupling constants in Hz are given in parenthesis.

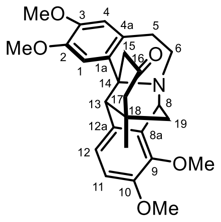

sinometumine E (**3**)

No.	δ_H, nat. (CD_3OD)[5]	δ_H, synt. ($CDCl_3$)	δ_C, nat. (CD_3OD)[5]	δ_C, synt. ($CDCl_3$)
1	6.95, s	6.68, s	110.8	108.3
1a	-	-	133.9	132.0
2	-	-	148.4	147.2
3	-	-	149.0	146.6
4	6.25, s	6.14, s	112.6	111.1
4a		-	130.2	128.8
5	2.28, m;<br>2.08, m	2.25, ddd (15.7, 11.0, 4.3);<br>2.10 – 2.01, m	26.9	25.8
6	3.08, m; 3.02, m	3.08, ddd (13.4, 10.9, 3.6);<br>2.99, dt (13.4, 4.3)	48.4	47.1
8	4.06, dd (3.8, 1.9)	4.03, dd (3.9, 1.9)	54.2	52.5
8a	-	-	135.9	134.4
9	-	-	143.6	142.1
10	-	-	152.4	150.8
11	6.59, d (8.0)	6.42, d (8.0)	111.3	109.7
12	6.57, d (8.0)	6.45, d (8.0)	123.9	122.3
12a	-	-	132.9	131.0
13	3.37, s	3.07, s	54.0	53.42
14	-	-	64.6	62.9
15	2.99, d (13.9);<br>2.62, dd (13.9, 2.3)	2.73, d (14.0);<br>2.70, d (14.0)	54.3	53.36
16	-	-	213.2	209.1
17	2.68, d (14.0);<br>2.39, dd (14.0, 2.3)	2.48, d (14.3);<br>2.44, d (14.3)	56.0	55.3
18	-	-	37.3	35.9
19	2.01, dd (12.4, 3.8);<br>1.10, dd (12.4, 1.9)	2.10 – 2.01, m;<br>1.08, dd (12.5, 1.9)	45.0	43.5
2-OMe	3.85, s	3.86, s	57.0	56.2
3-OMe	3.66, s	3.66, s	56.2	55.48/55.46*
9-OMe	3.80, s	3.78, s	61.6	61.0
10-OMe	3.73, s	3.69, s	56.2	55.48/55.46*
18-Me	0.81, s	0.79, s	31.8	31.4

* the exact assignment cannot be performed due to the low difference in the chemical shifts

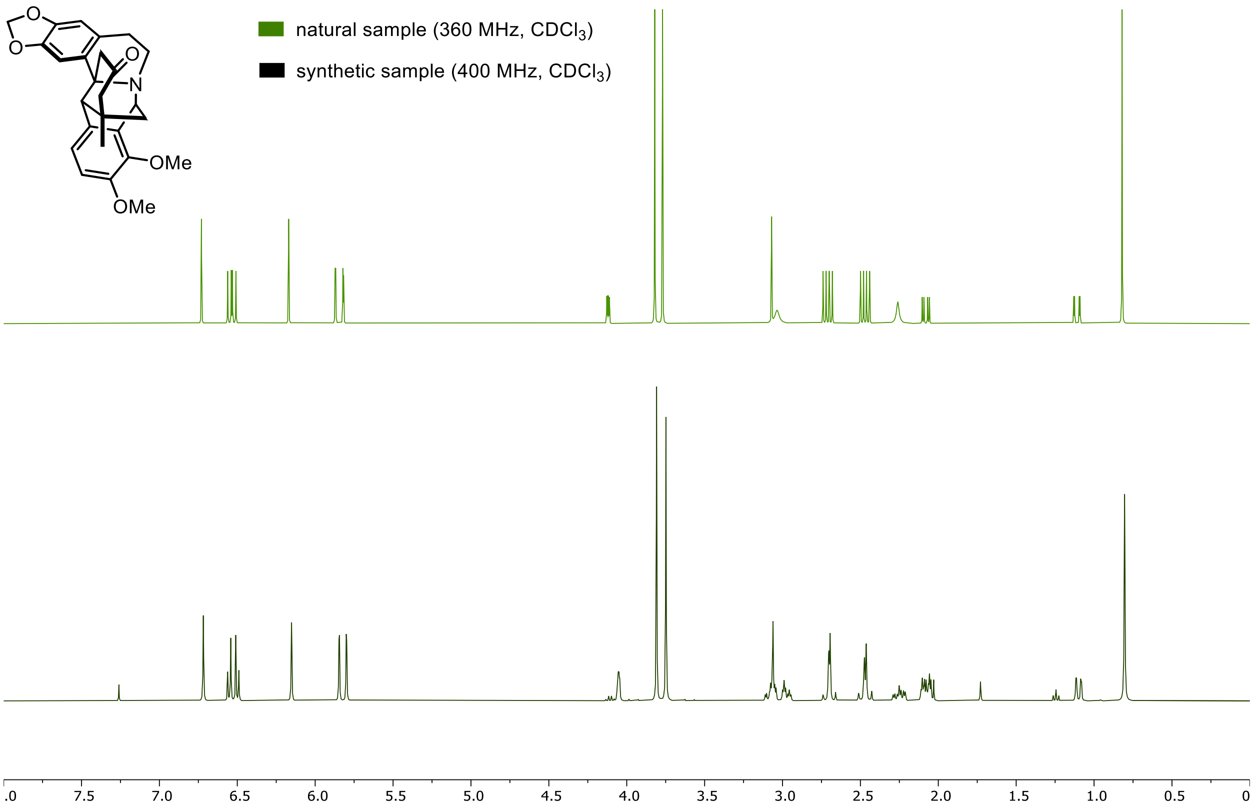

Figure S4. Visual comparison of ^{1}H NMR data of synthetic and natural compound **1** (the spectrum of the natural sample was simulated using the data reported previously[1]).

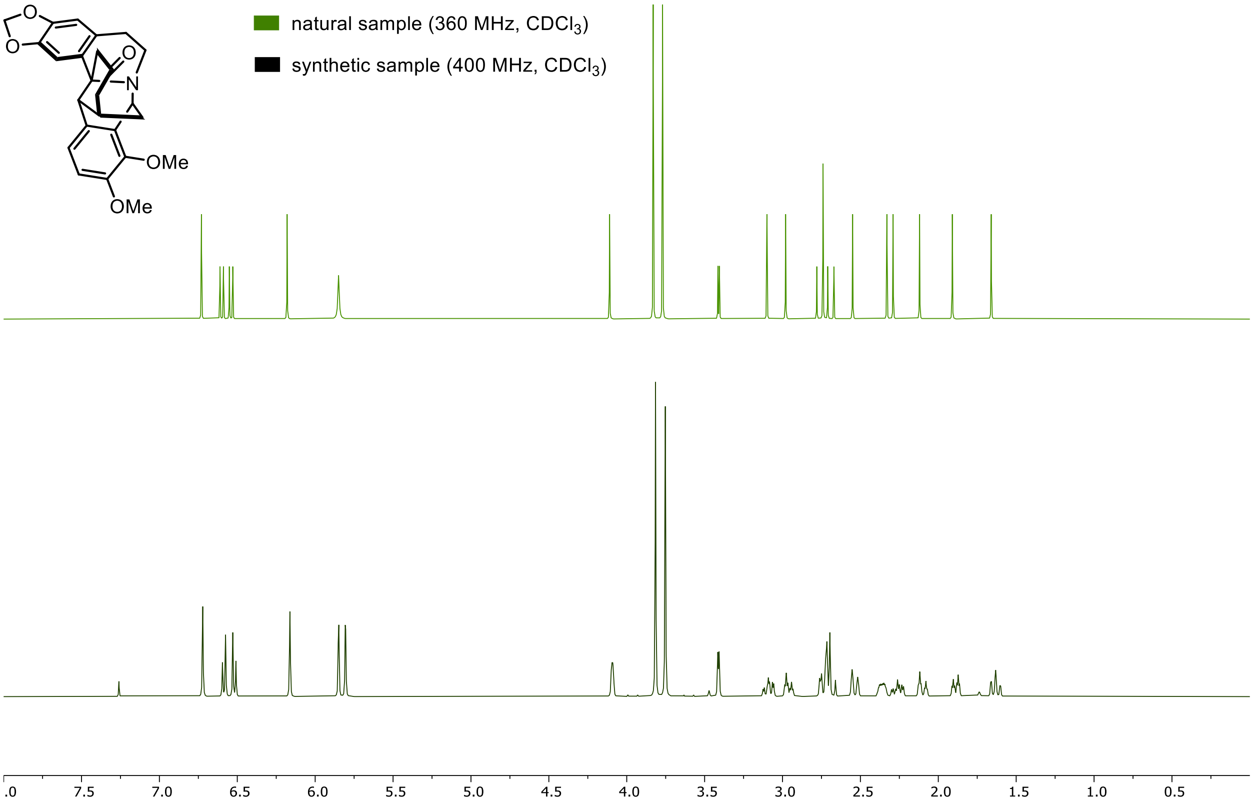

Figure S5. Visual comparison of ^{1}H NMR data of synthetic and natural compound **2** (the spectrum of the natural sample was simulated using the data reported previously[3]).

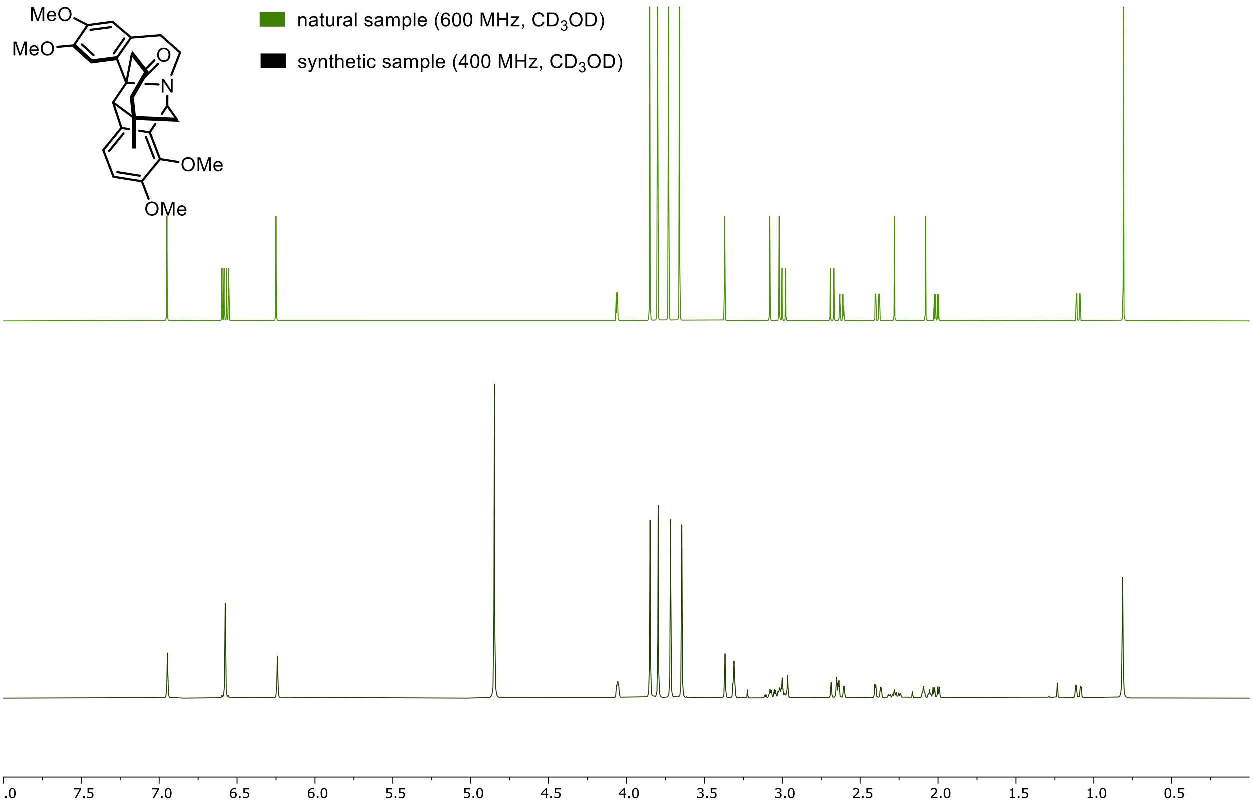

Figure S6. Visual comparison of ^{1}H NMR data of synthetic and natural compound **3** (the spectrum of the natural sample was simulated using the data reported previously[5]).

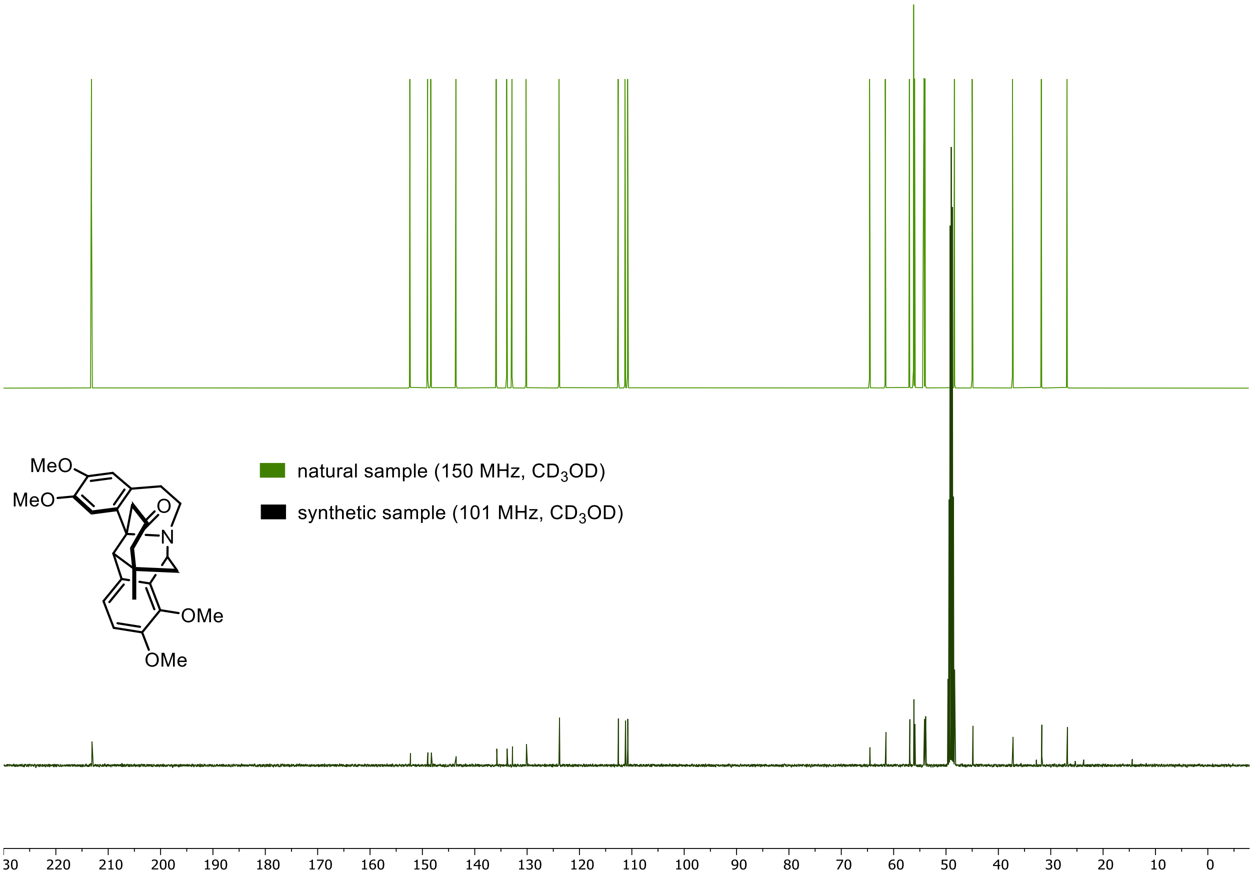

Figure S7. Visual comparison of ^{13}C NMR data of synthetic and natural compound **3** (the spectrum of the natural sample was simulated using the data reported previously[5]).

4. Crystallographic data

The diffraction data of single crystals of **1** (fa0932) and **12** (av_123) samples were obtained on Bruker D8 VENTURE Kappa Duo PHOTONIII by IμS micro-focus sealed tube either MoKα (λ= 0.71073Å) or CuKα (λ= 1.54178 Å) at low temperature of the crystal preserved by Cryostream Cooler. The structures were solved by direct methods (XT)[6] and refined by full matrix least squares based on F^2 (SHELXL2019)[7]. The hydrogen atoms on carbon were fixed into idealized positions (riding model) and assigned temperature factors either $H_{iso}(H) = 1.2\ U_{eq}$(pivot atom) or $H_{iso}(H) = 1.5$ for methyl moieties.

The crystals of both samples contain solvating molecules, in **12** (av_123), the pentane molecules are disordered into two positions and even more both are placed near inversion center of the symmetry space group giving rise the complicated structure. Therefor several restrictions were applied during refinement on geometry of the pentane, occupancy and displacement factors of its atoms. The solution of the disorder enables obtaining the structure of main molecules in reliable precision.

X-ray crystallographic data have been deposited with the Cambridge Crystallographic Data Centre (CCDC) can be obtained free of charge from the Centre via its website (www.ccdc.cam.ac.uk/structures/). The corresponding CCDC numbers are in Table S7.

Table S7. Crystal data, data collection, and refinement parameters for **1** and **12**.

Compound	**1** (fa0932)	**12** (av_123)
CCDC	2492814	2492813
Formula	$C_{26}H_{27}NO_5 \cdot 0.5(C_4H_8O_2)$	$2(C_{26}H_{29}NO_5) \cdot C_5H_{12}$
M.w.	477.54	943.15
Crystal system	Monoclinic	Monoclinic
Space group	*C*2/*c* (No.15)	*C*2/*c* (No.15)
a [Å]	29.368 (3)	28.200 (2)
b [Å]	8.9625 (8)	9.5621 (8)
c [Å]	21.5590 (19)	21.0799 (18)
α [°]	-	-
β [°]	123.851 (2)	122.326 (3)
γ [°]	-	-
Z	8	4
$V [Å^3]$	4712.6 (7)	4803.2 (7)
Temperature	120	100
D_x [g cm^{-3}]	1.346	1.304
Wavelength, Å	1.54178	0.71073
Crystal size [mm]	0.22 × 0.13 × 0.10	0.29 × 0.27 × 0.15
Crystal color, shape	Prism, colourless	Irregular, colourless
μ [mm^{-1}]	0.77	0.09
T_{min}, T_{max}	0.851, 0.924	0.975, 0.987
Measured reflections	32950	66870
Independent diffractions (R_{int}^a)	5060, (0.026)	5971, (0.025)
Observed diffract. [$I>2\sigma(I)$]	4843	5587
No. of parameters	348	376
R^b	0.037	0.039
$wR(F^2)$ for all data	0.097	0.105
GOF^c	1.04	1.03
Residual electron density [e/Å^3]	0.33, -0.23	0.38, -0.28

^{a} $R_{int} = \Sigma|F_o^2 - F_{o,mean}^2|/\Sigma F_o^2$;

^{b} $R(F) = \Sigma||F_o| - |F_c||/\Sigma|F_o|$; $wR(F^2) = [\Sigma(w(F_o^2 - F_c^2)^2)/(\Sigma w(F_o^2)^2)]^{½}$;

^{c} GOF $= [\Sigma(w(F_o^2 - F_c^2)^2)/(N_{diffrs} - N_{params})]^{½}$

5. Copies of NMR spectra

9,10-Dimethoxy-13-methyl-5,6,8,12b,13,14-hexahydro-8,13-methano[1,3]dioxolo[4',5':6,7]isoquinolino[1,2-*e*]phenanthridin-15(16*H*)-one (karachine, 1)

^{1}H NMR (400 MHz, $CDCl_3$)

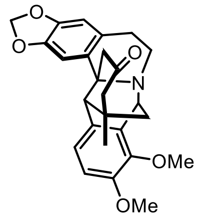

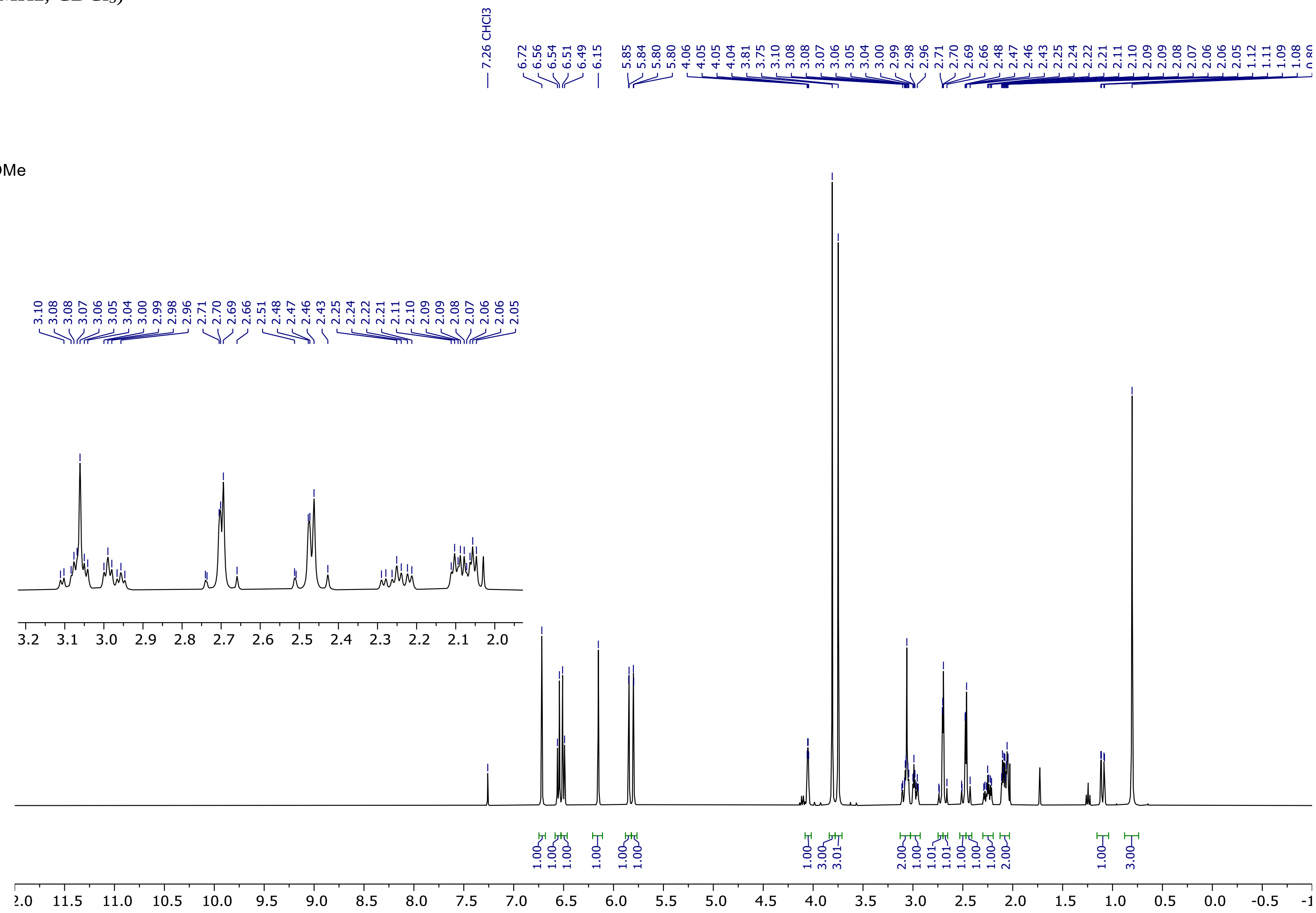

^{1}H NMR (400 MHz, CD_3OD)

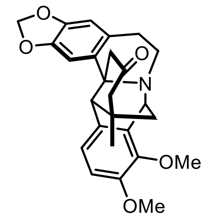

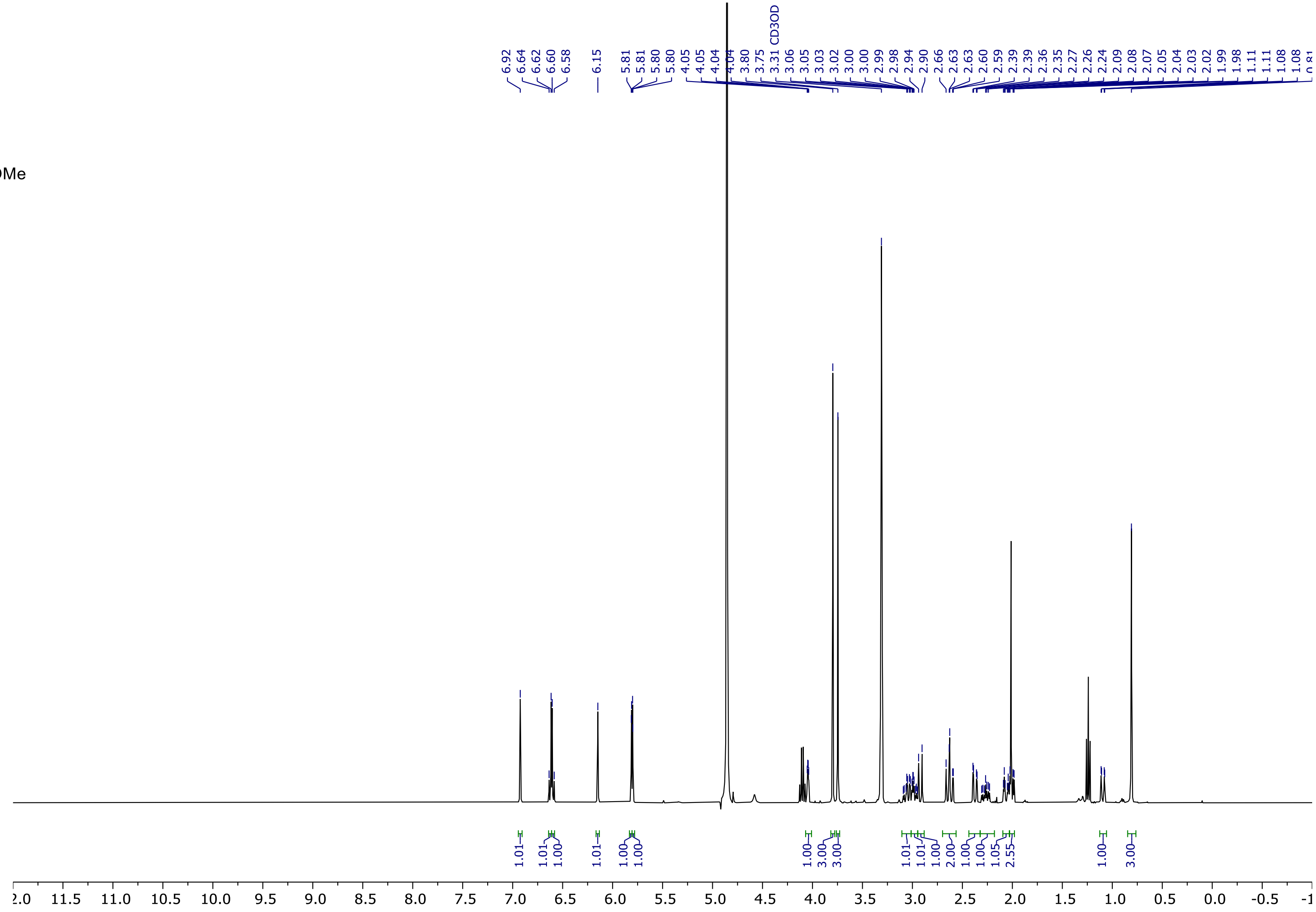

$^{13}C\{^{1}H\}$ NMR (101 MHz, $CDCl_3$)

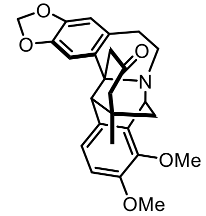

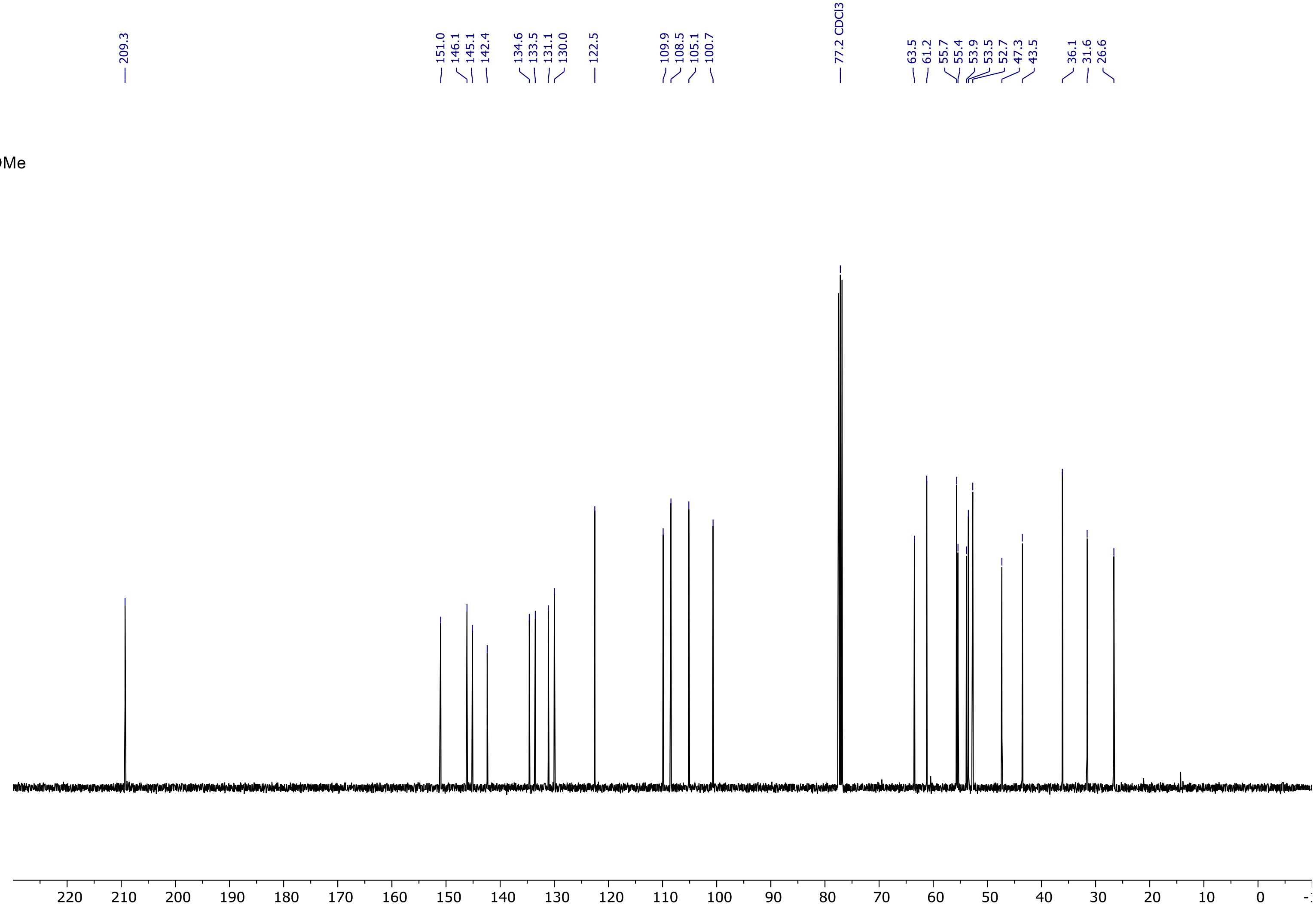

^{1}H–^{1}H COSY NMR ($CDCl_3$)

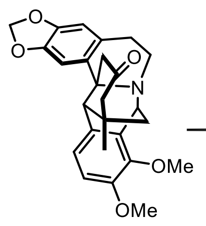

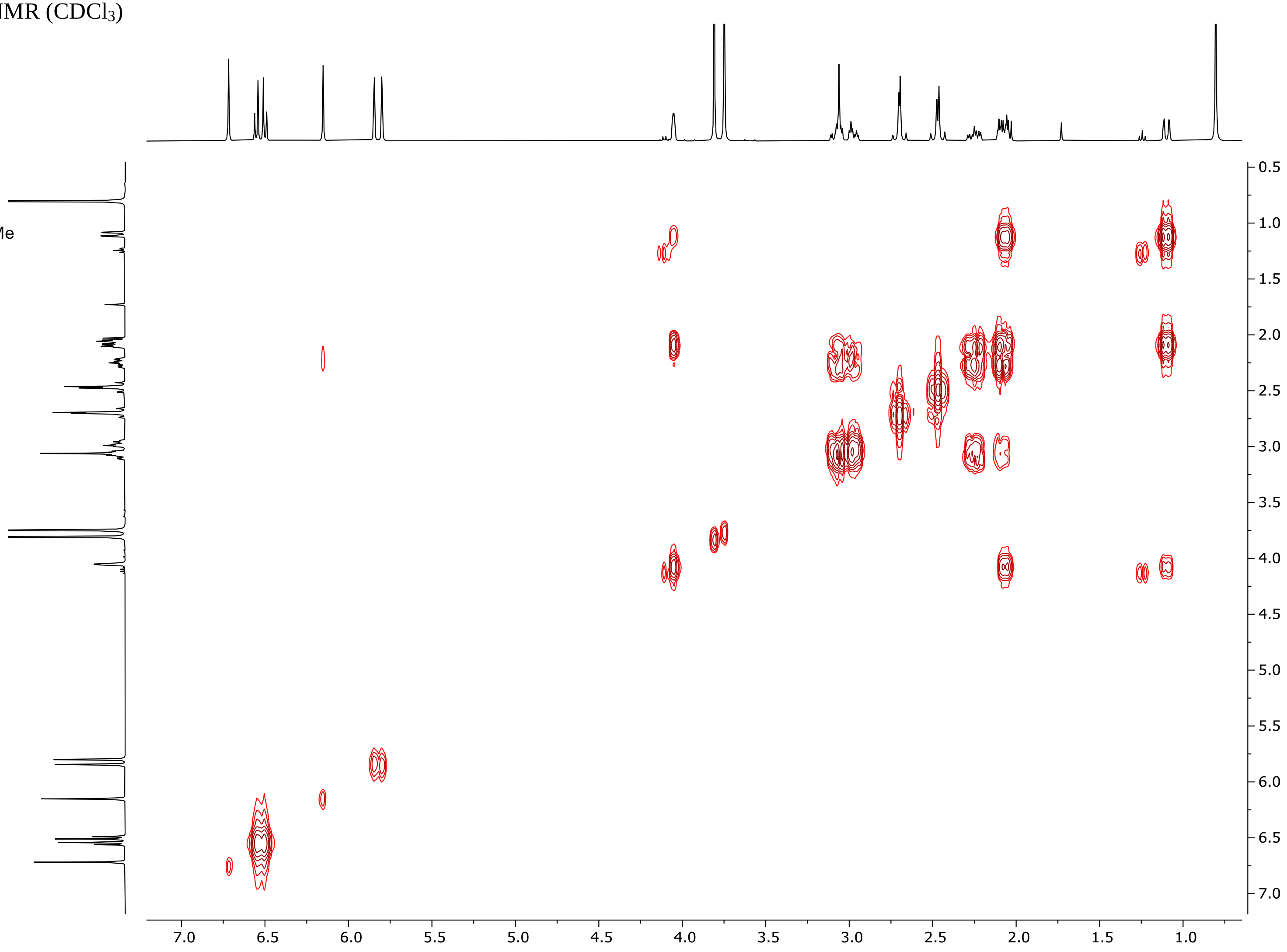

^{1}H–^{13}C HSQC NMR ($CDCl_3$)

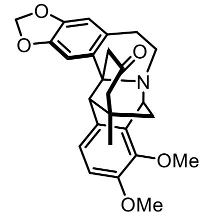

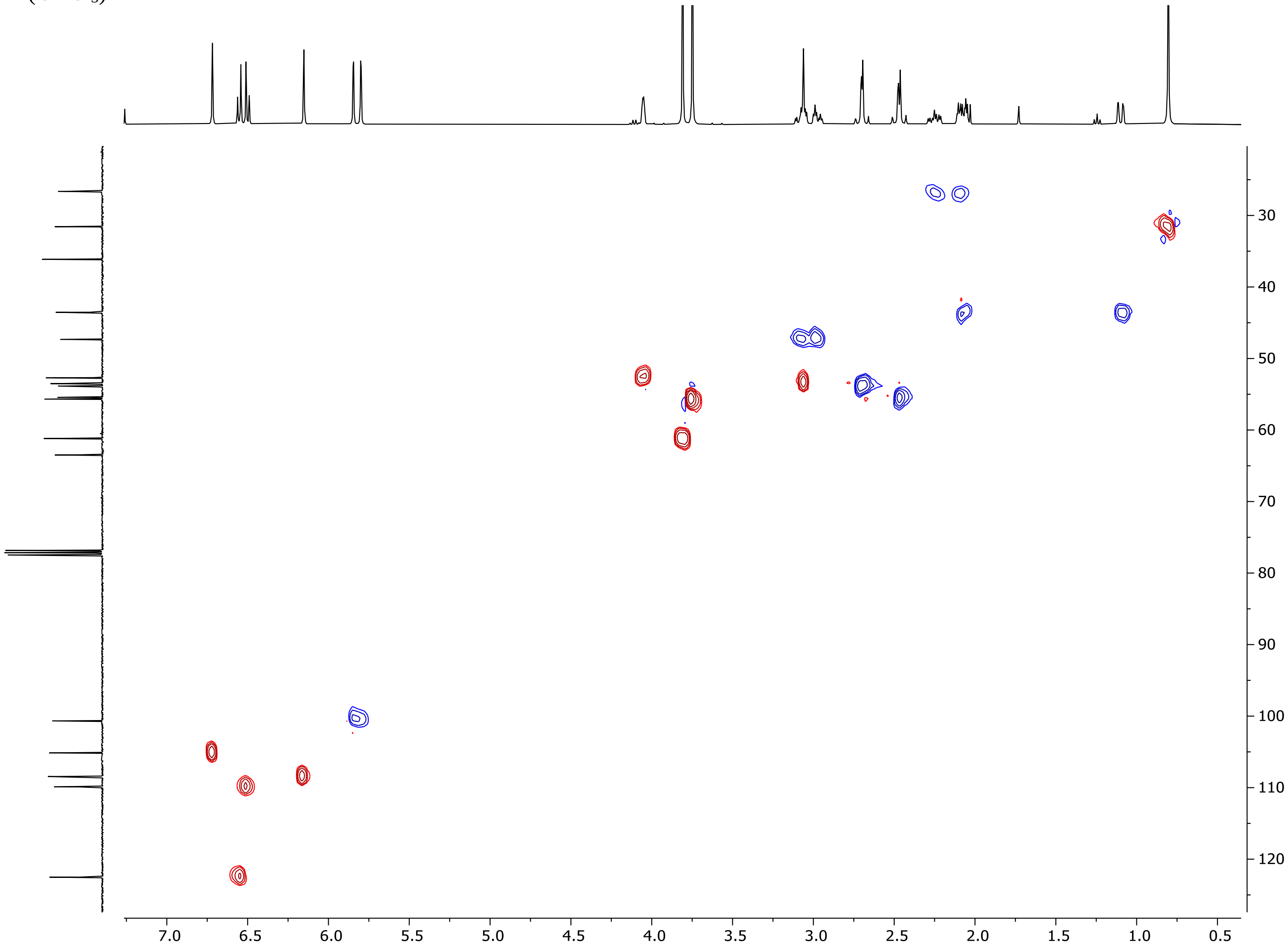

^{1}H–^{13}C HMBC NMR ($CDCl_3$)

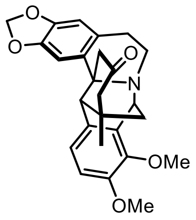

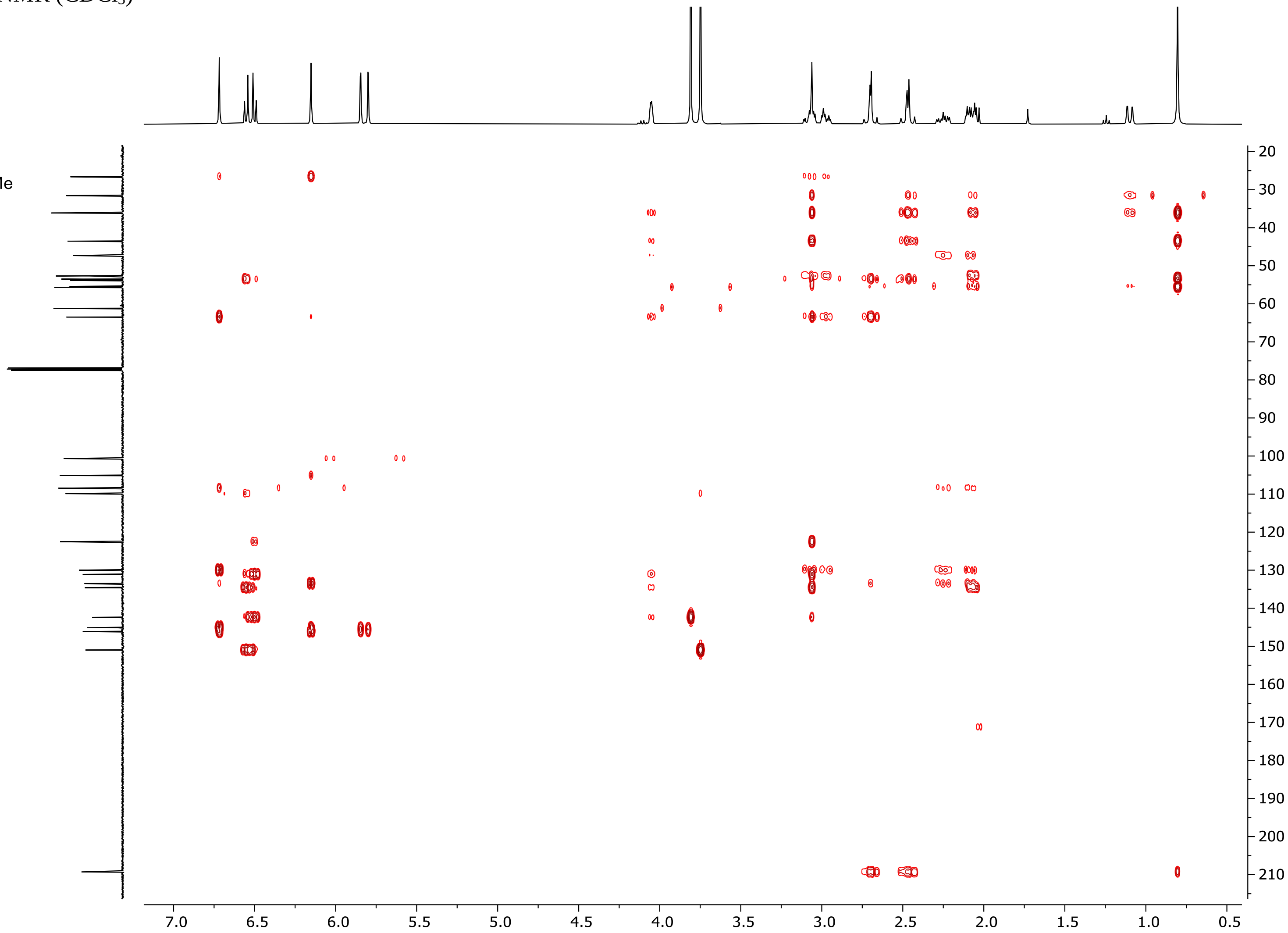

9,10-Dimethoxy-5,6,8,12b,13,14-hexahydro-8,13-methano[1,3]dioxolo[4',5':6,7]isoquinolino[1,2-*e*]phenanthridin-15(16*H*)-one (valachine, 2)

^{1}H NMR (400 MHz, $CDCl_3$)

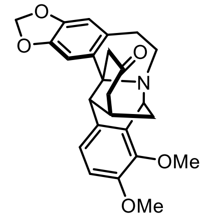

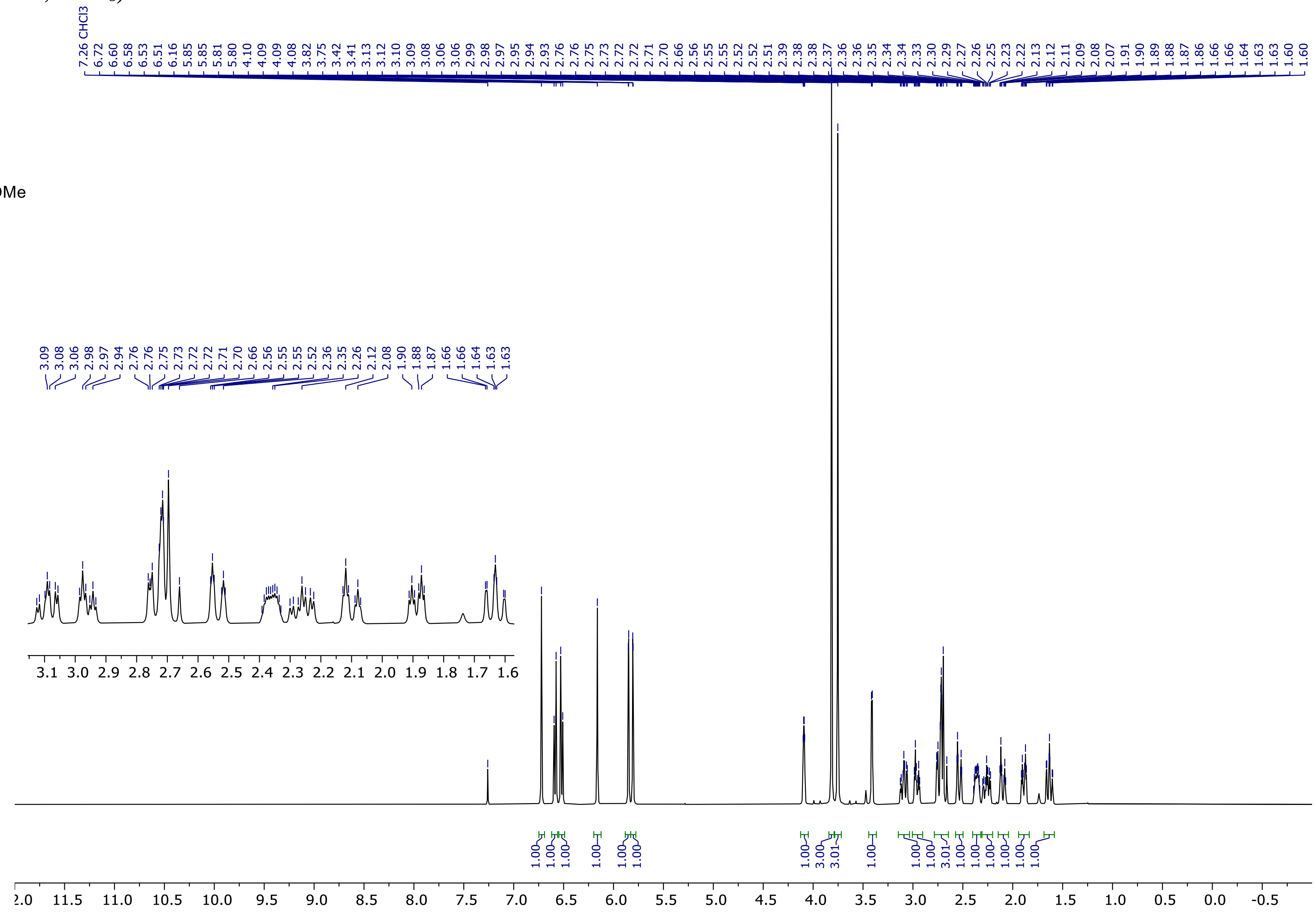

^{1}H NMR (400 MHz, CD_3OD)

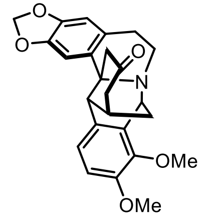

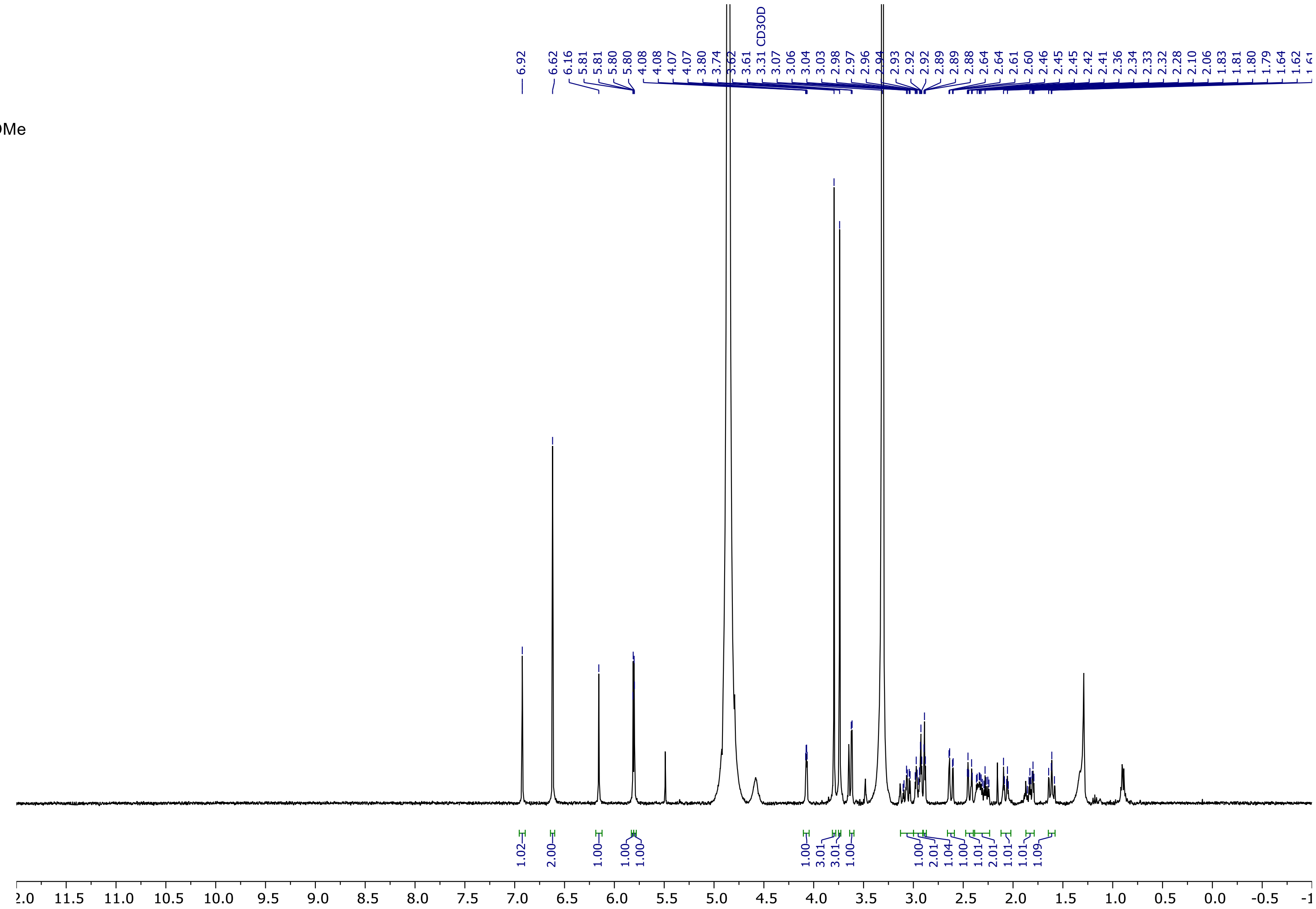

$^{13}C\{^1H\}$ NMR (101 MHz, $CDCl_3$)

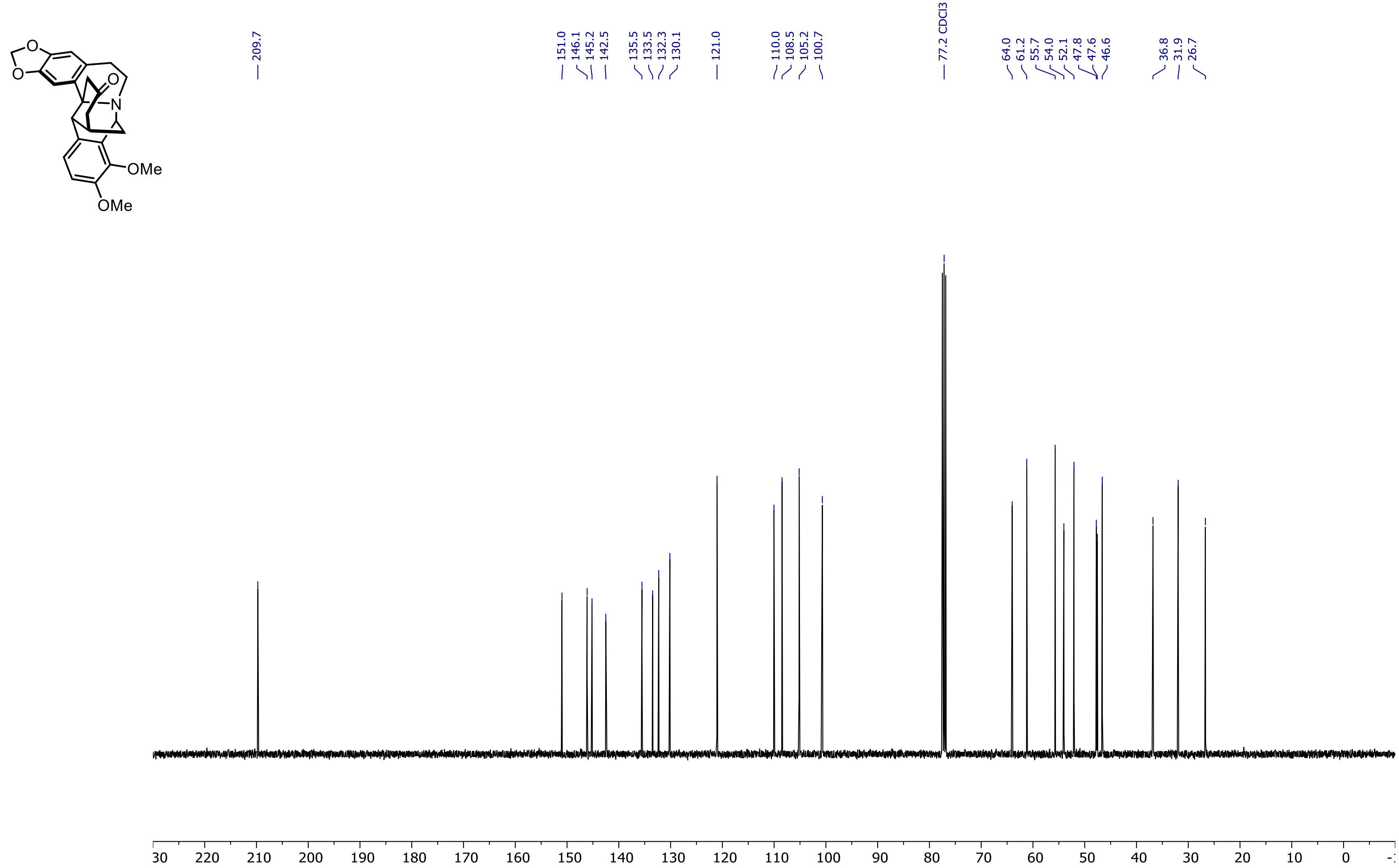

^{1}H–^{1}H COSY NMR ($CDCl_3$)

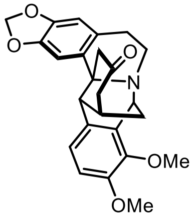

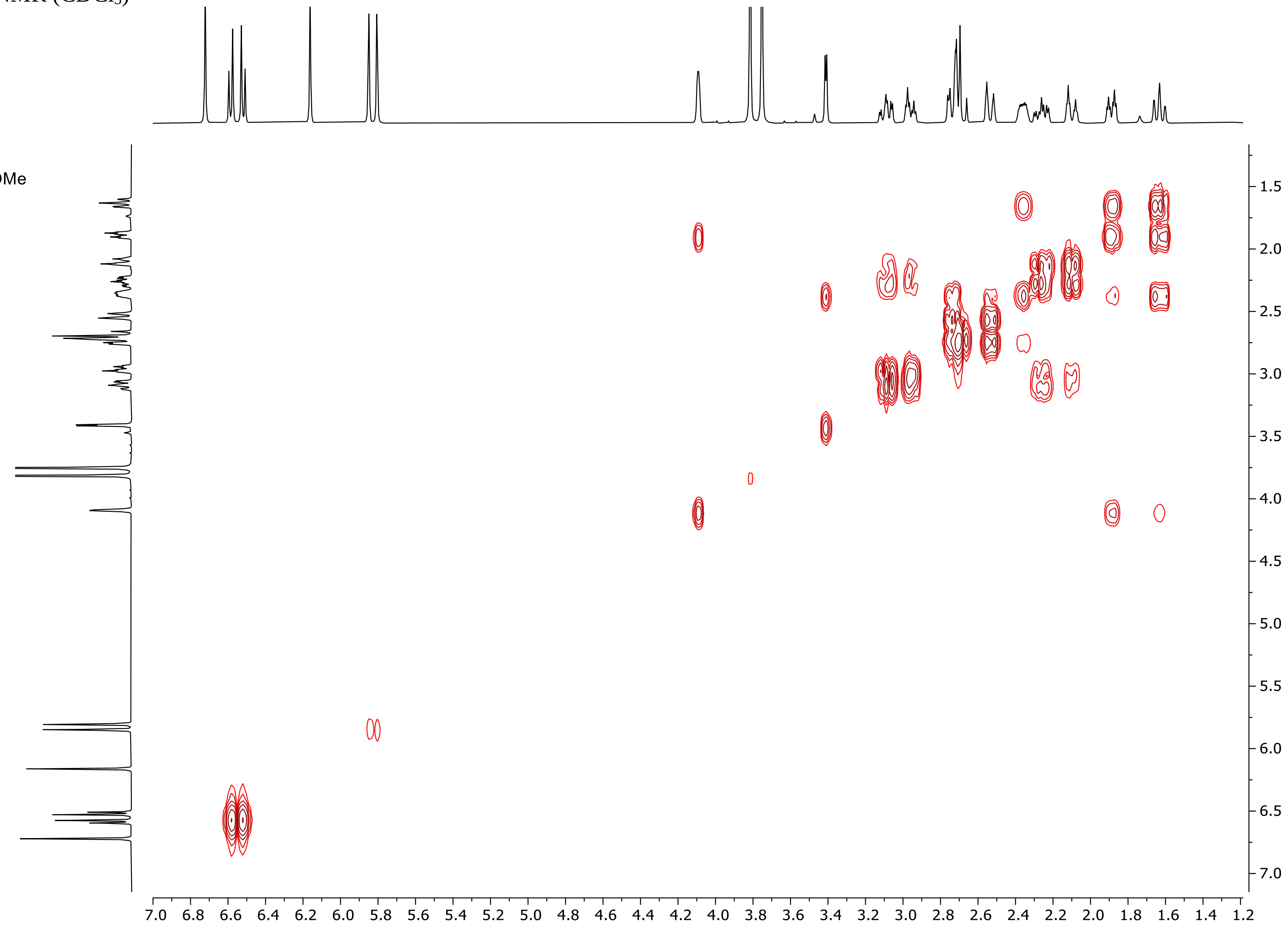

^{1}H–^{13}C HSQC NMR ($CDCl_3$)

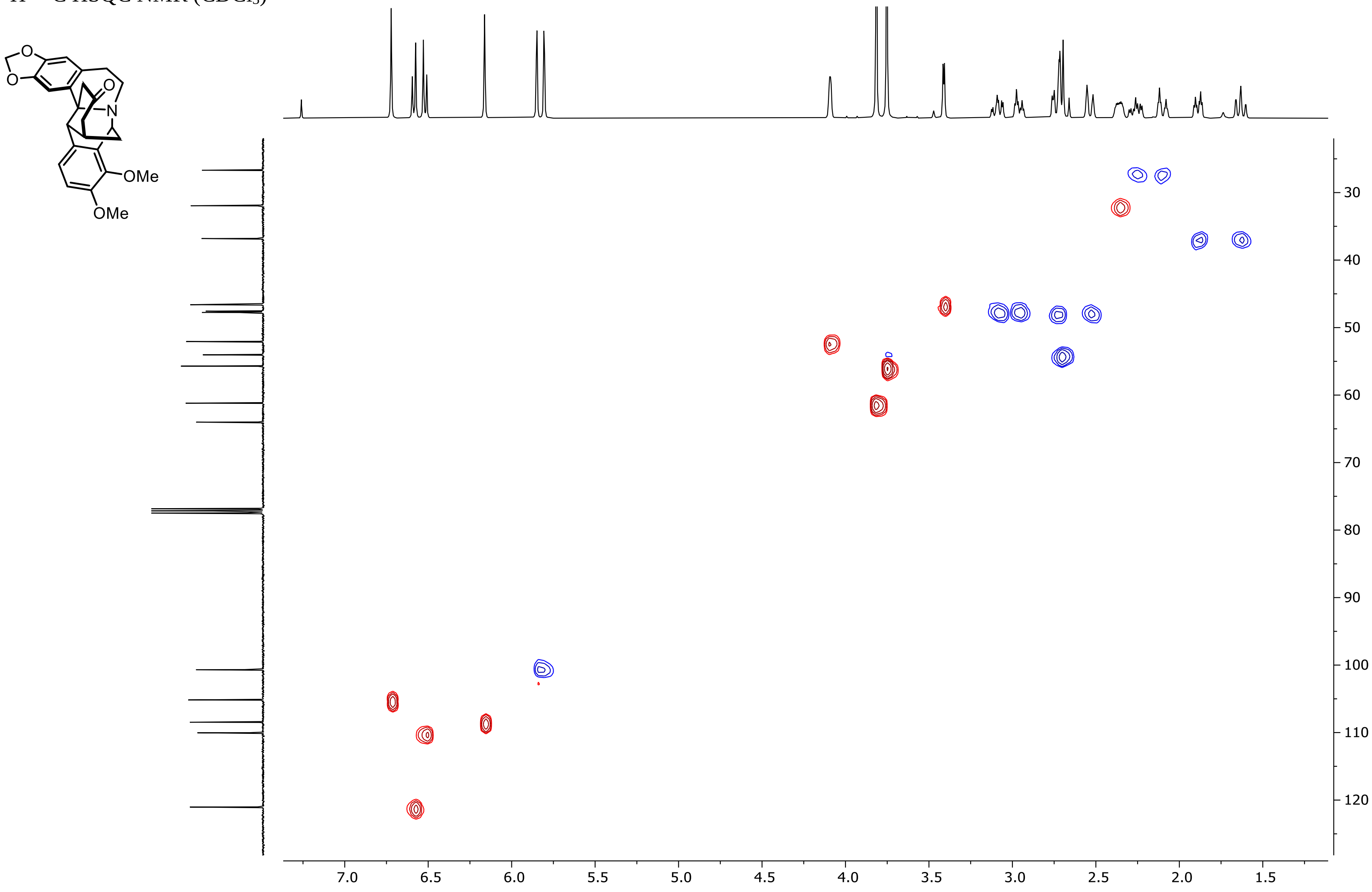

^{1}H–^{13}C HMBC NMR ($CDCl_3$)

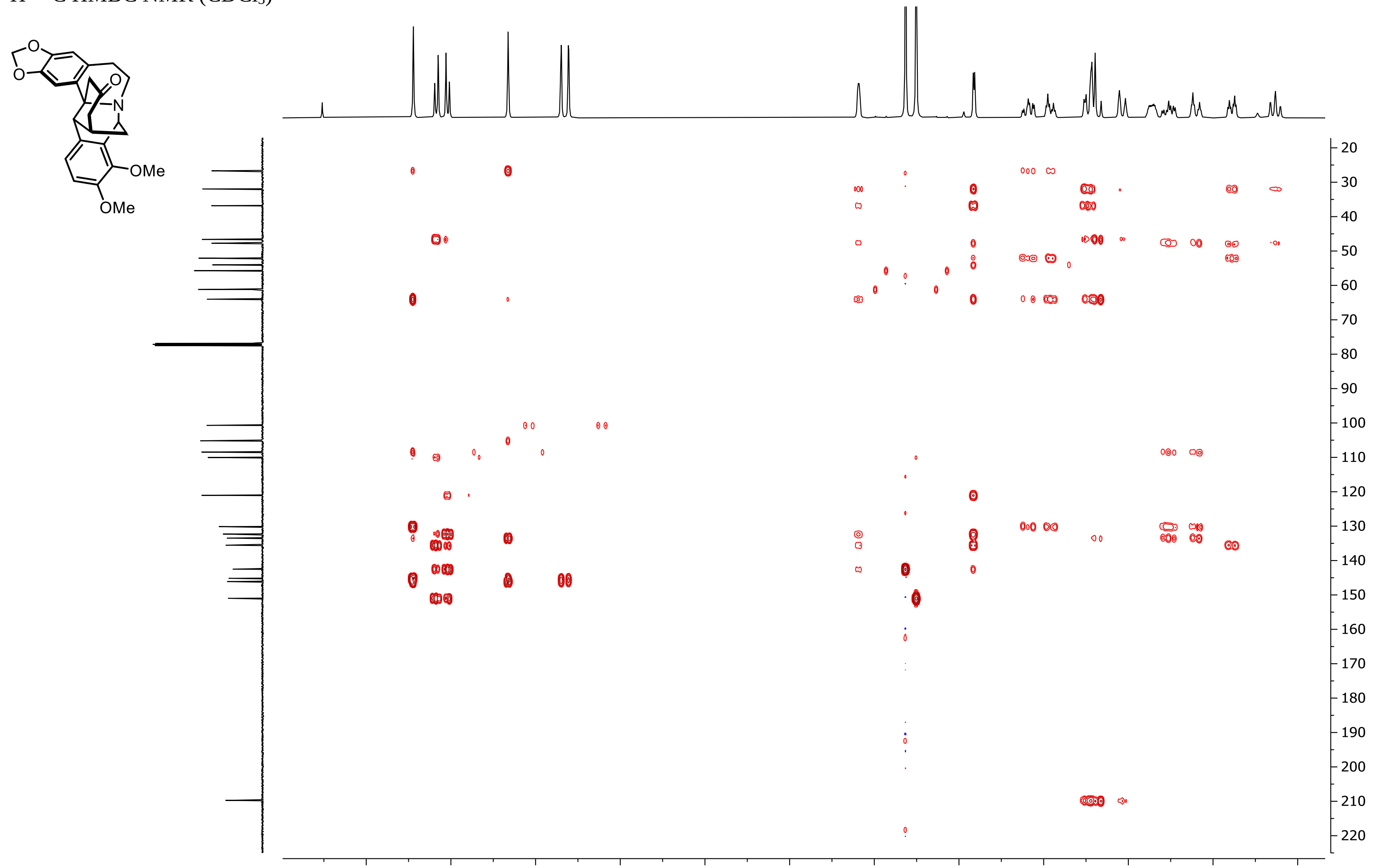

2,3,9,10-Tetramethoxy-13-methyl-5,6,8,12b,13,14-hexahydro-8,13-methanoisoquinolino[1,2-*e*]phenanthridin-15(16*H*)-one (sinometumine E, 3)

^{1}H NMR (400 MHz, $CDCl_3$)

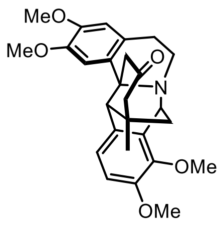

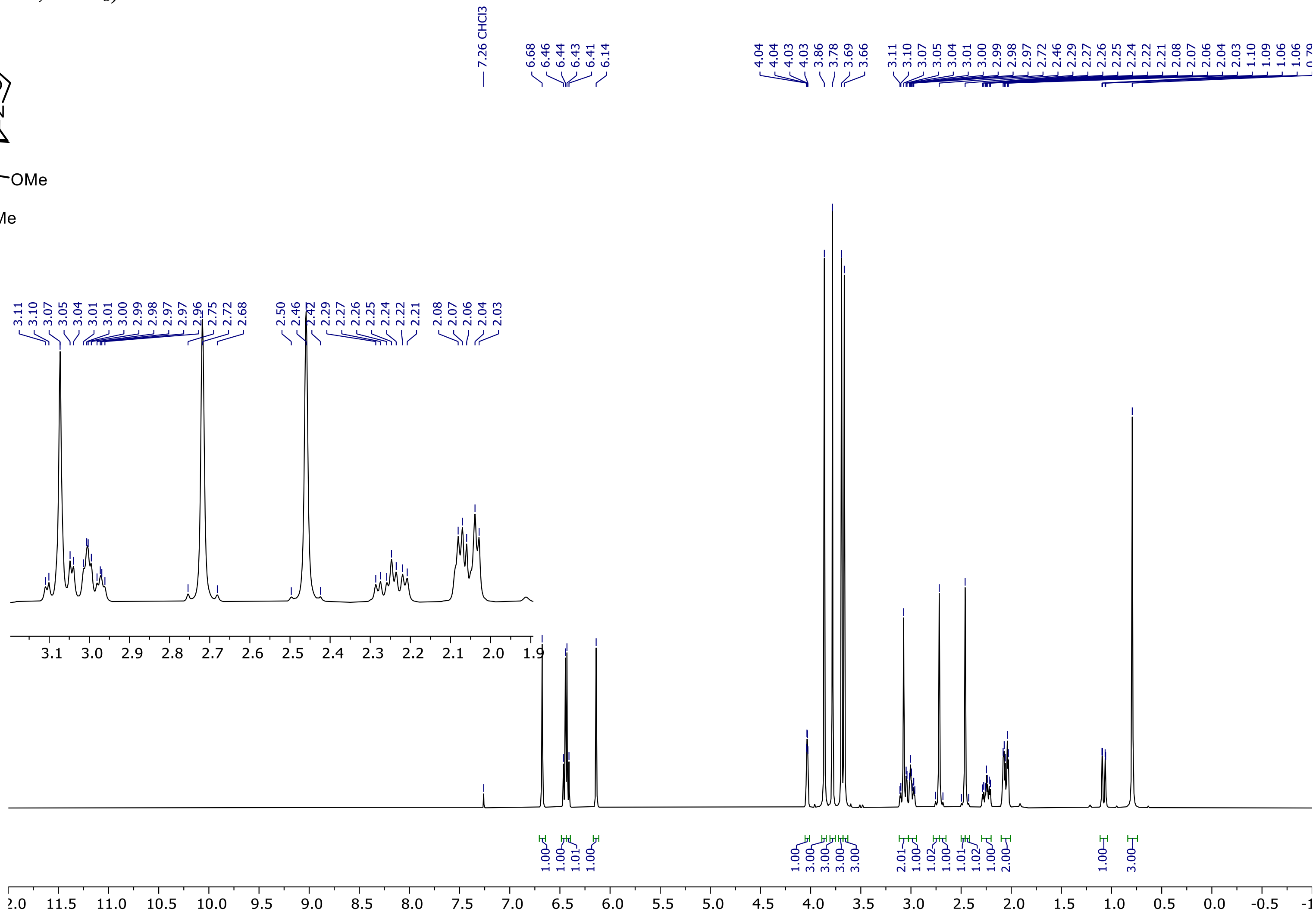

^{1}H NMR (400 MHz, CD_3OD)

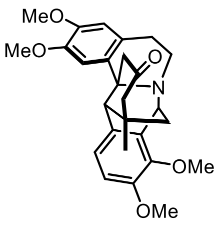

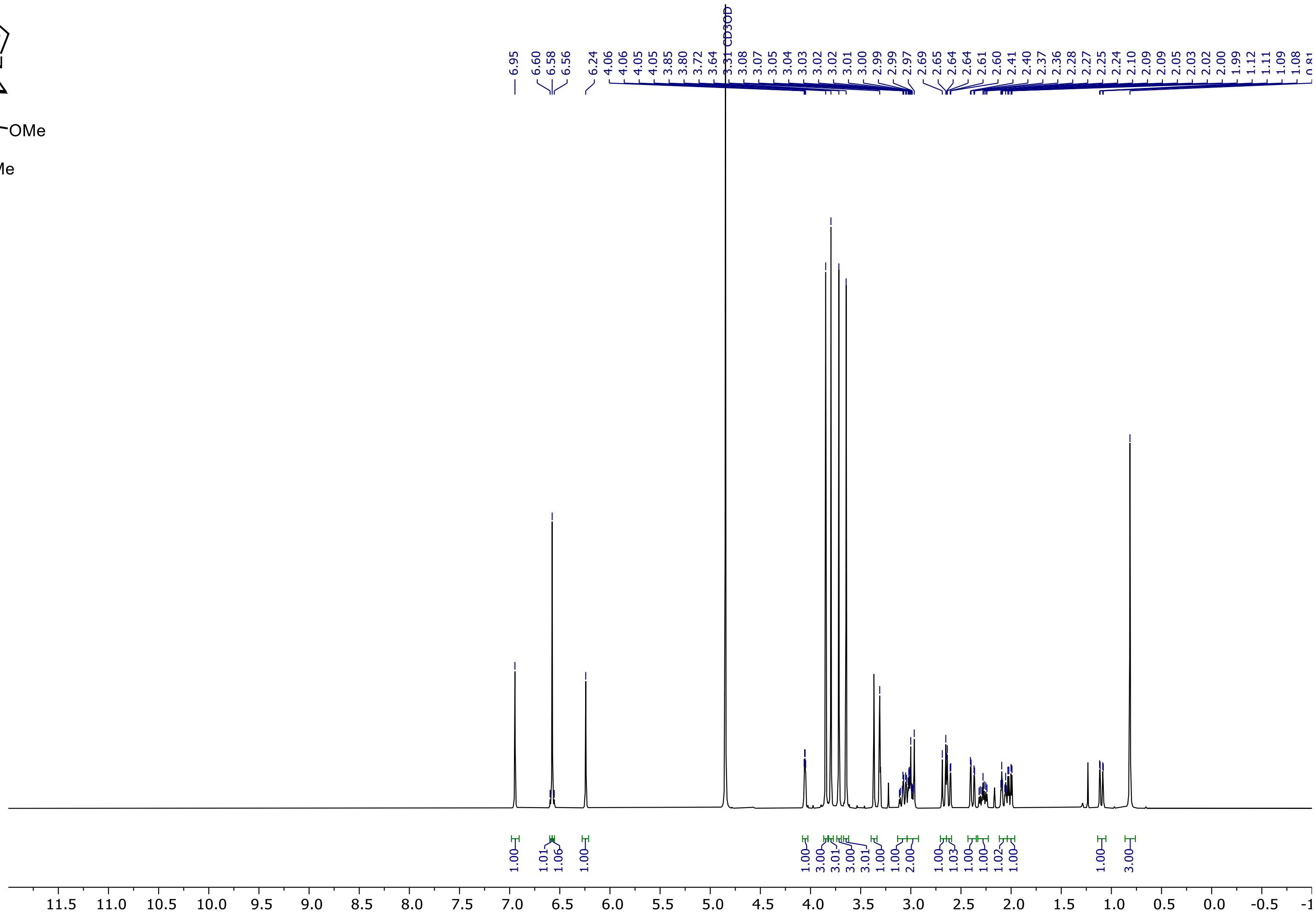

$^{13}C\{^1H\}$ NMR (101 MHz, $CDCl_3$)

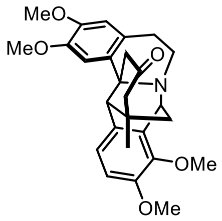

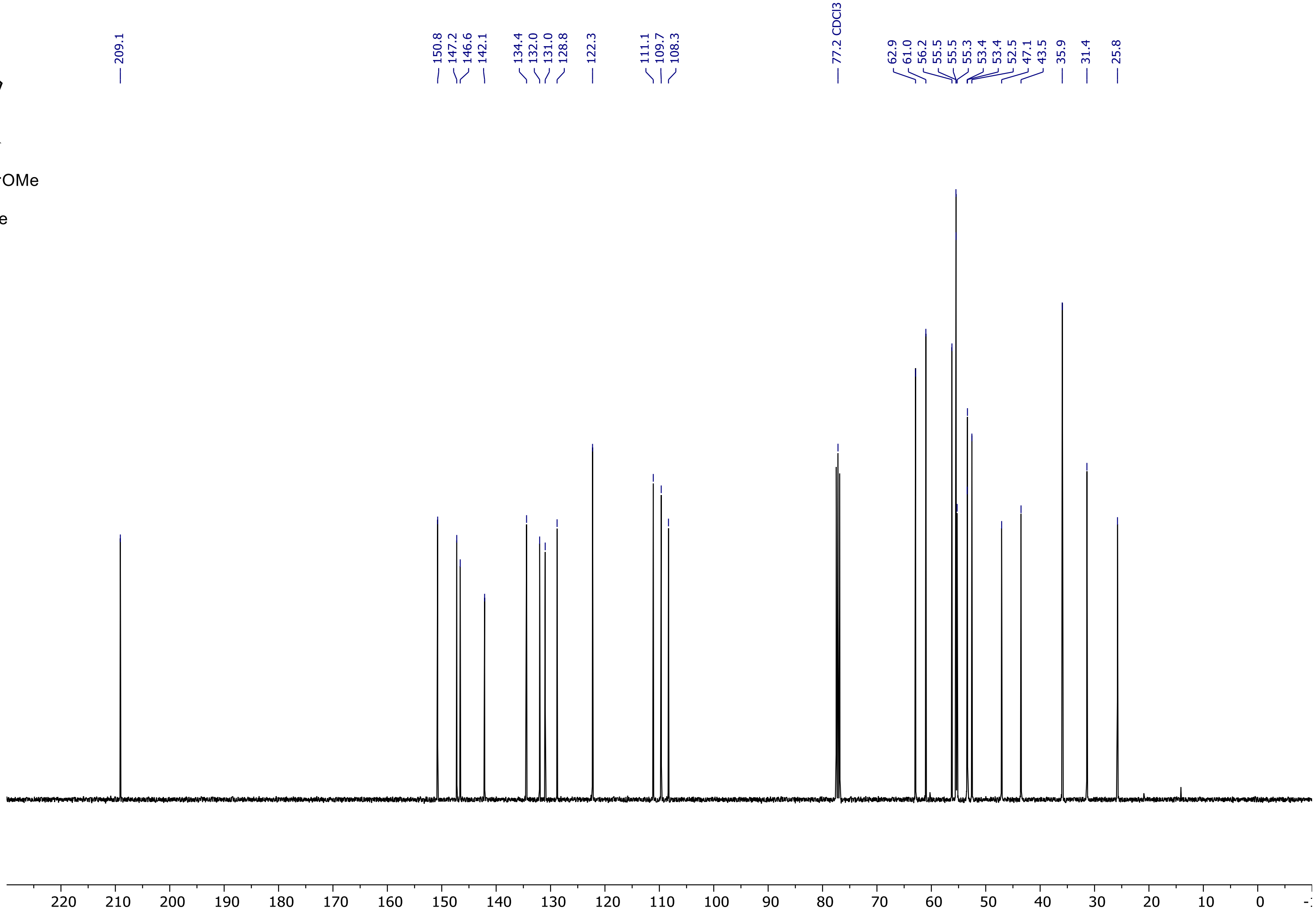

$^{13}C\{^{1}H\}$ NMR (101 MHz, CD_3OD)

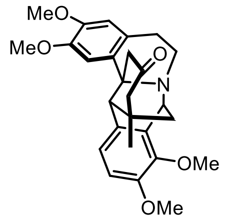

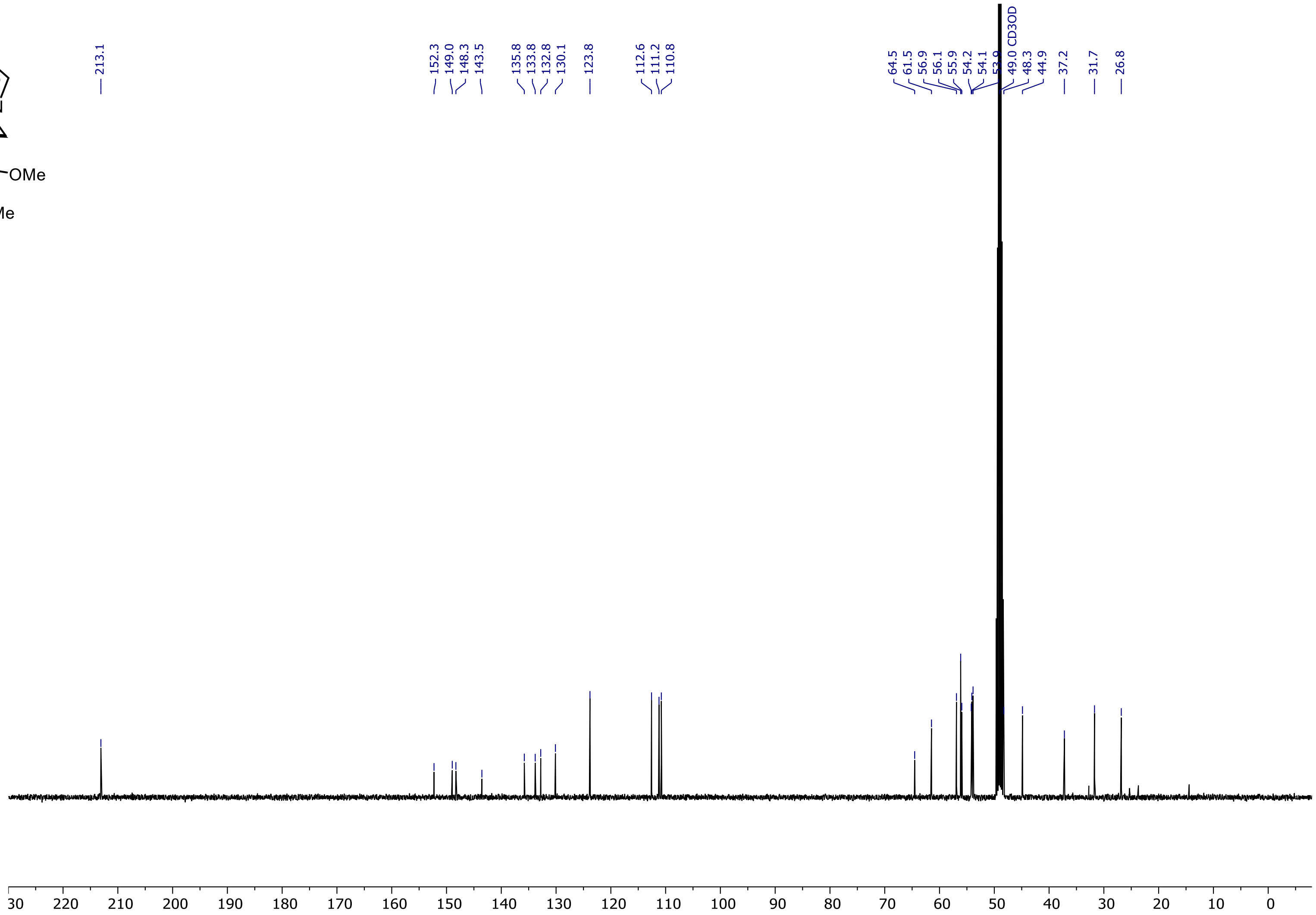

^1H–^1H COSY NMR ($CDCl_3$)

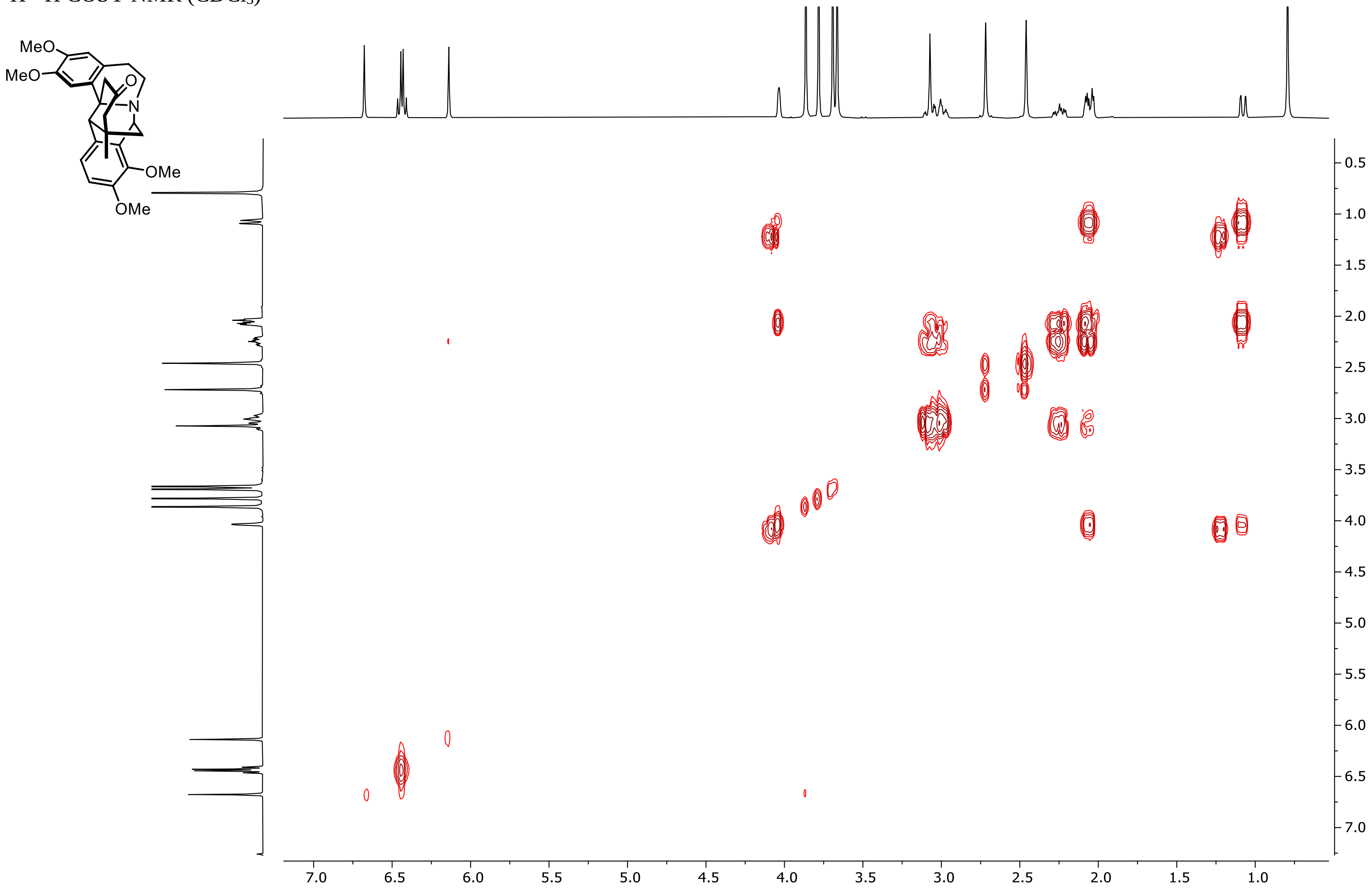

^{1}H–^{13}C HSQC NMR ($CDCl_3$)

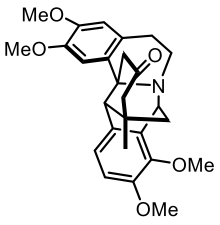

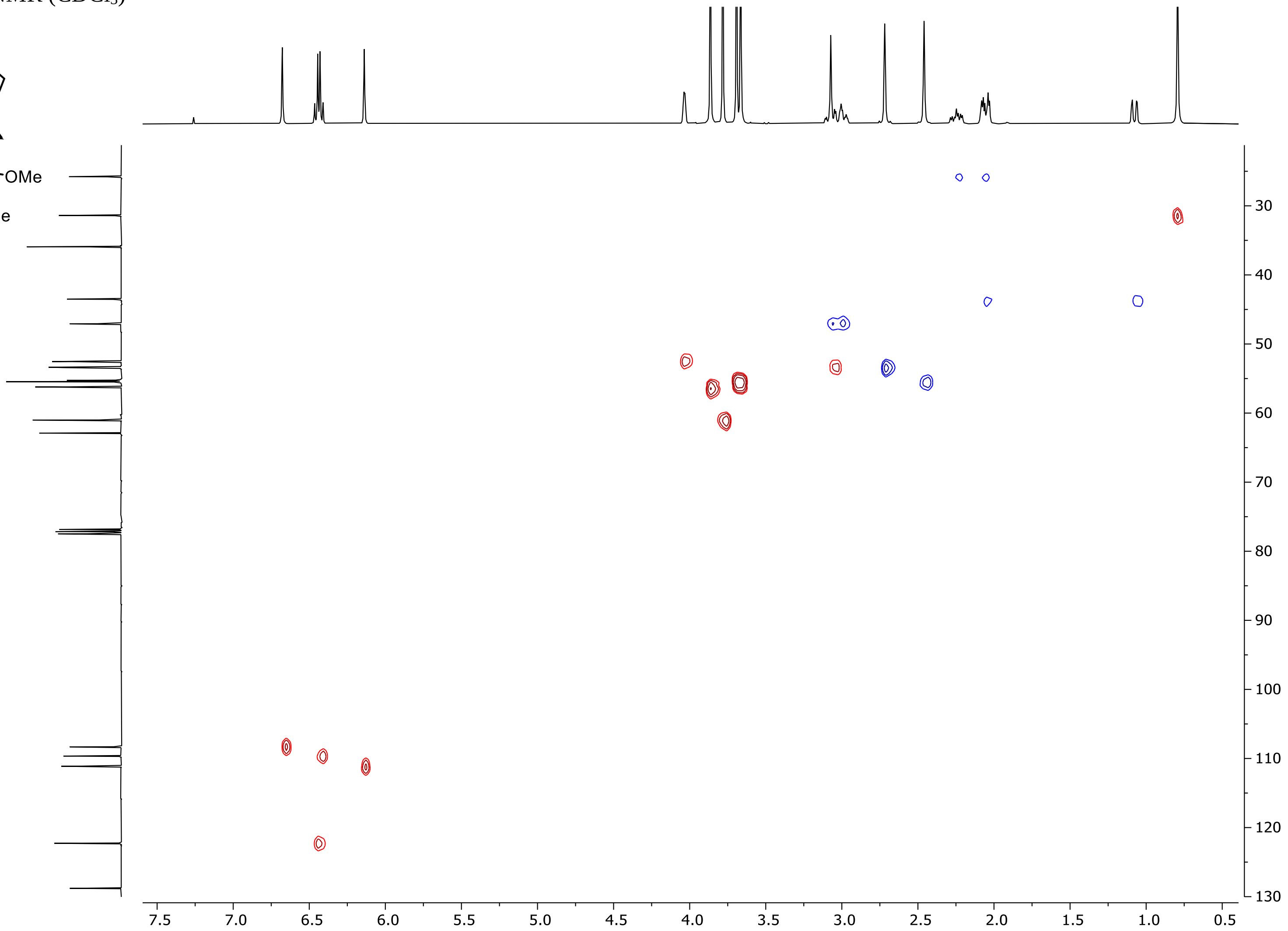

^1H–^{13}C HMBC NMR ($CDCl_3$)

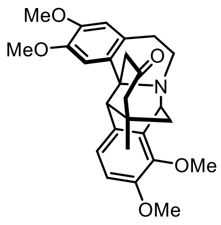

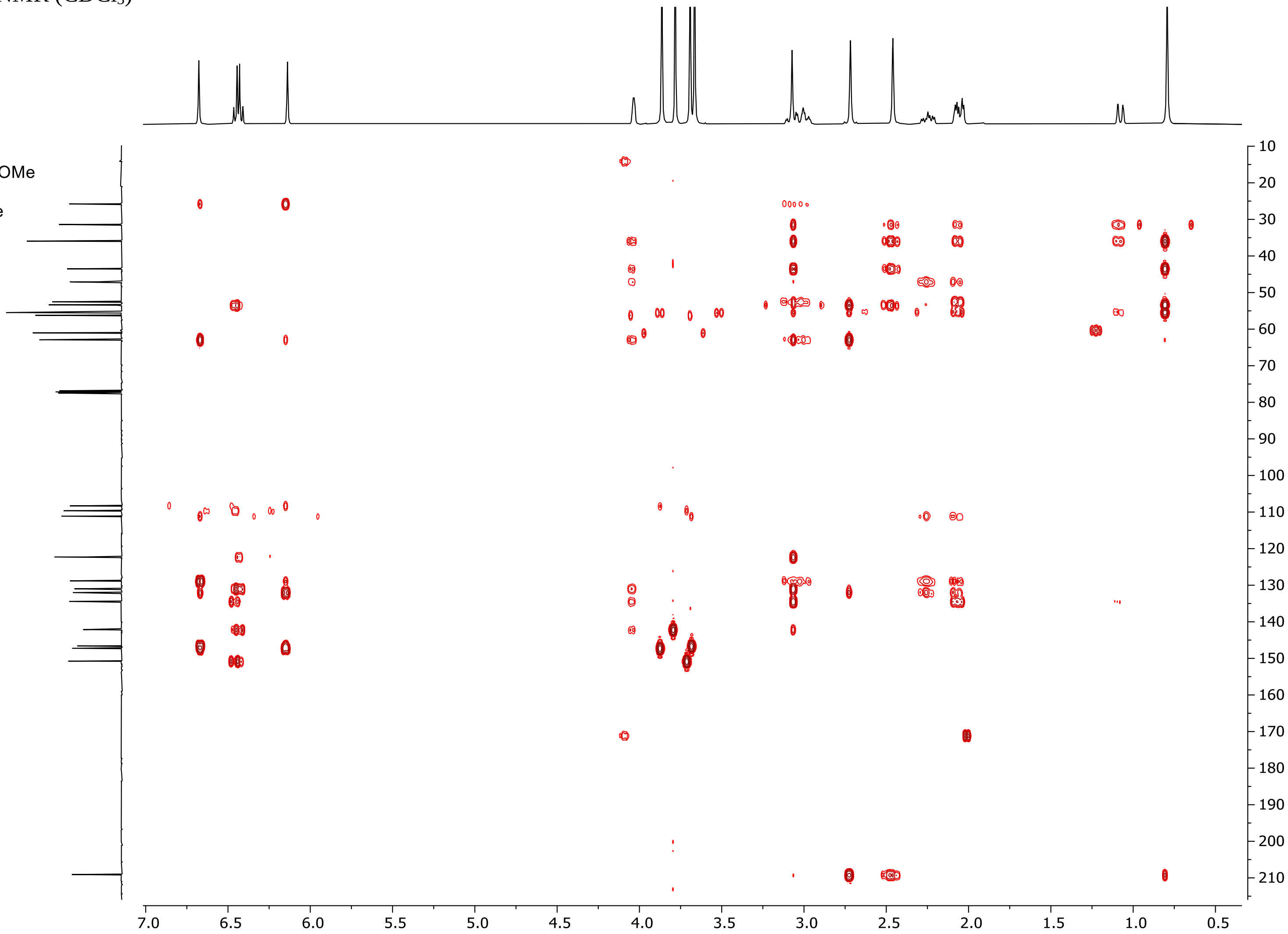

9,10-Dimethoxy-13-methyl-5,6,8,12b,13,14,15,16-octahydro-8,13-methano[1,3]dioxolo[4',5':6,7]isoquinolino[1,2-*e*]phenanthridin-15-ol (dihydrokarachine, 12)

^{1}H NMR (400 MHz, $CDCl_3$)

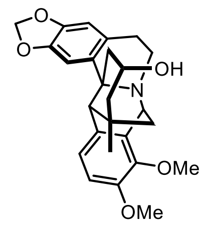

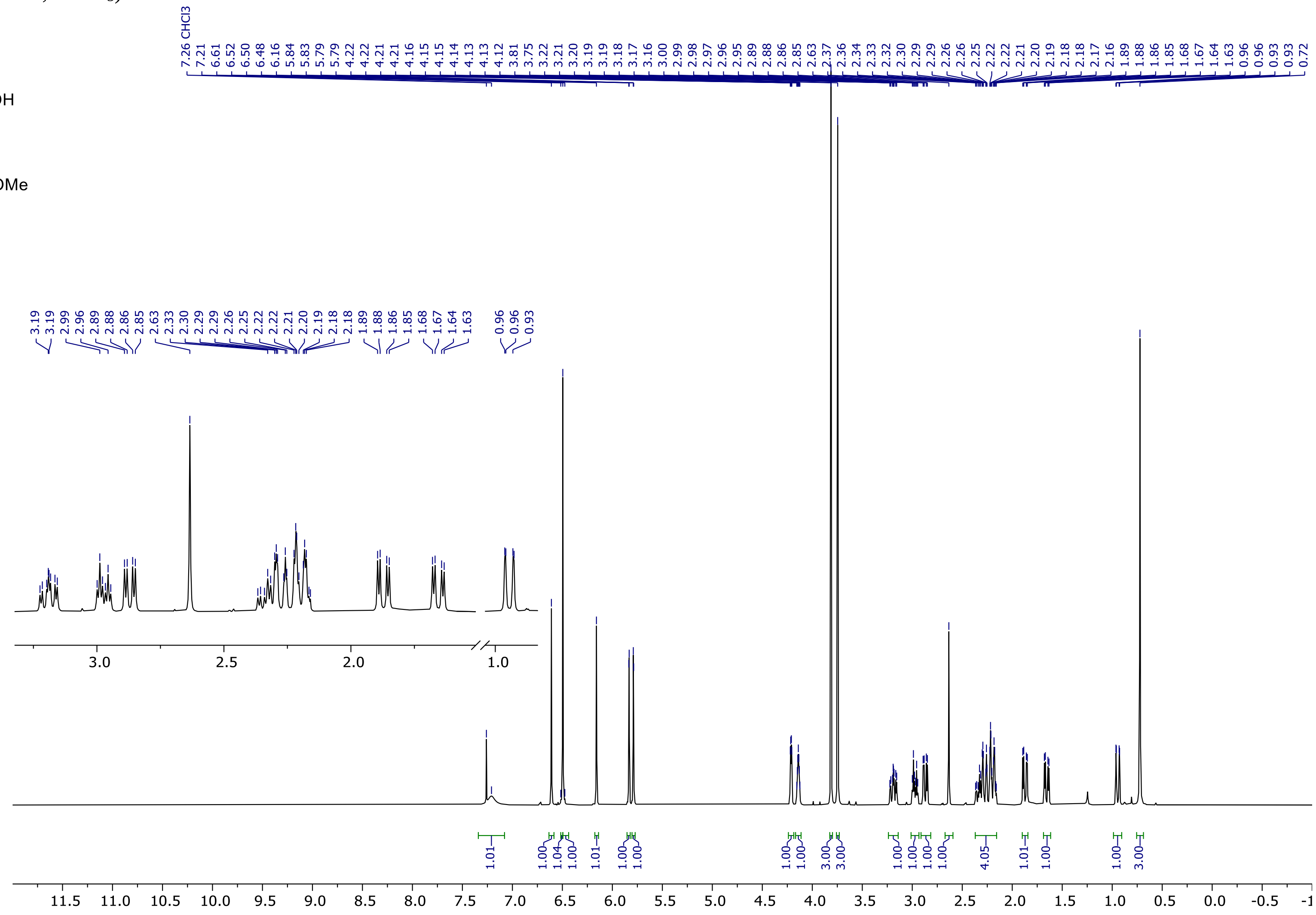

$^{13}C\{^{1}H\}$ NMR (101 MHz, $CDCl_3$)

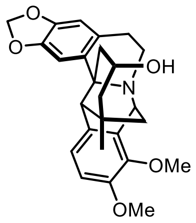

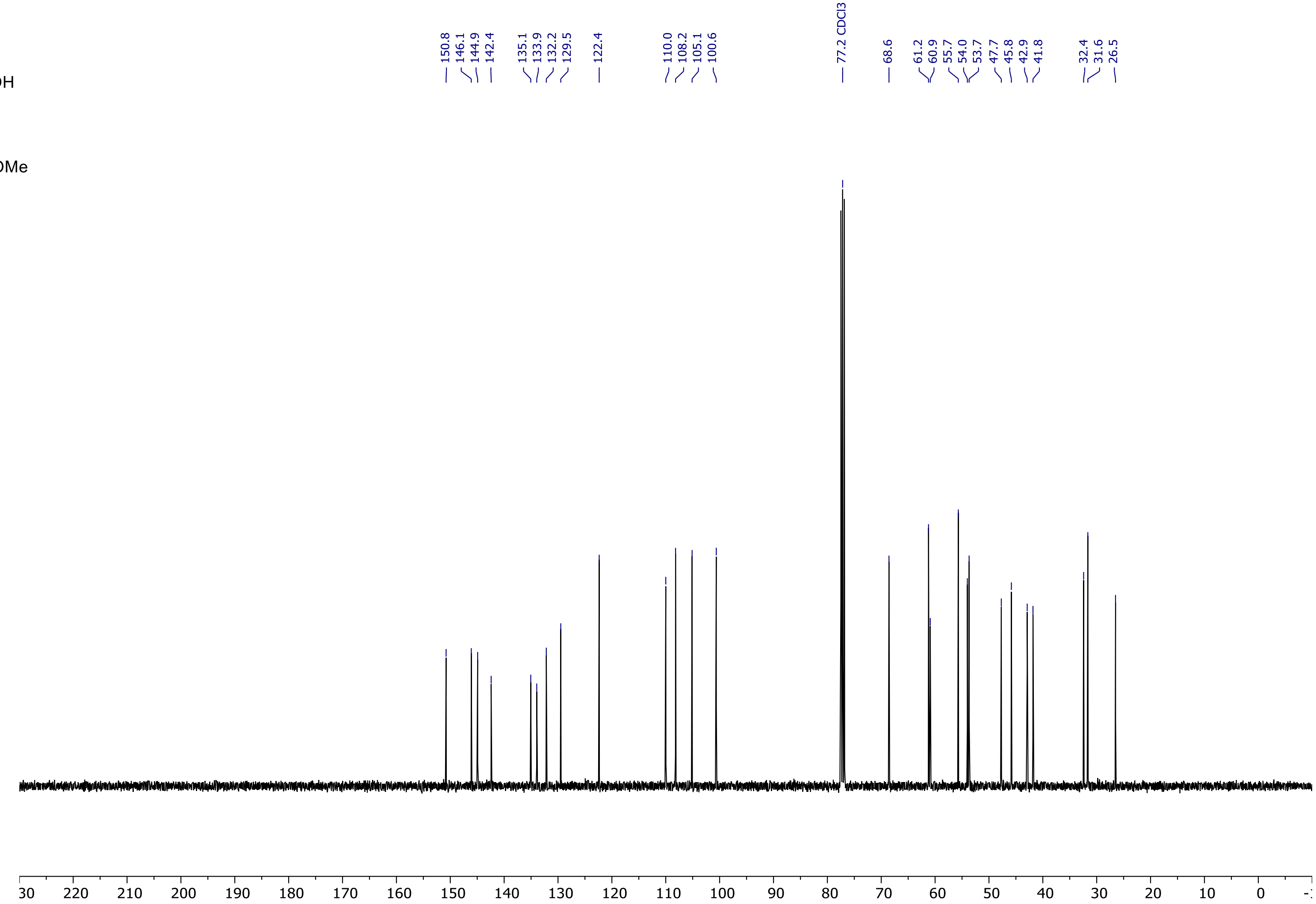

^{1}H–^{1}H COSY NMR ($CDCl_3$)

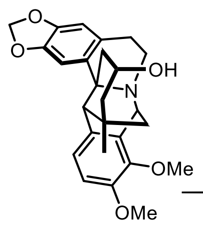

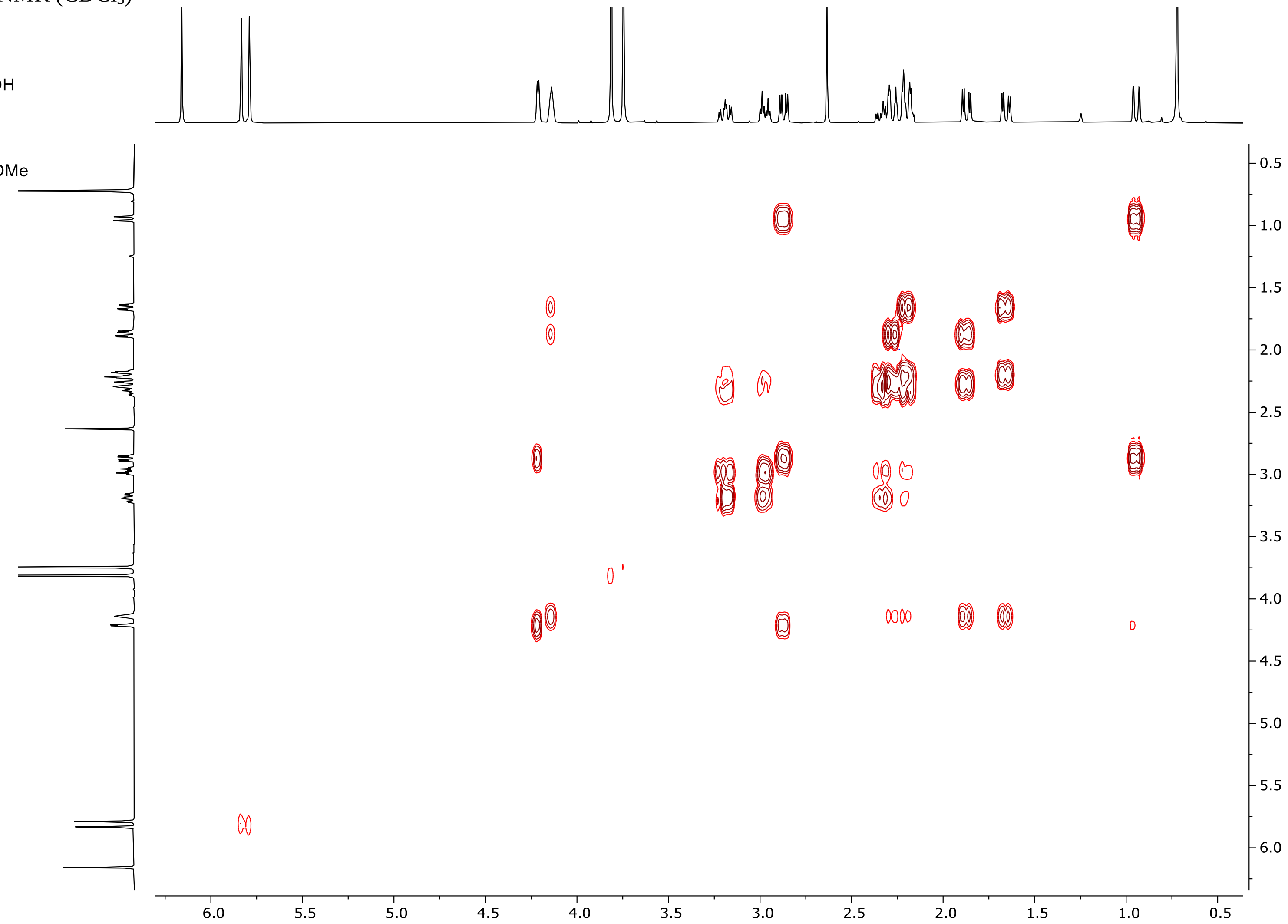

^{1}H–^{13}C HSQC NMR ($CDCl_3$)

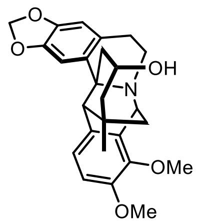

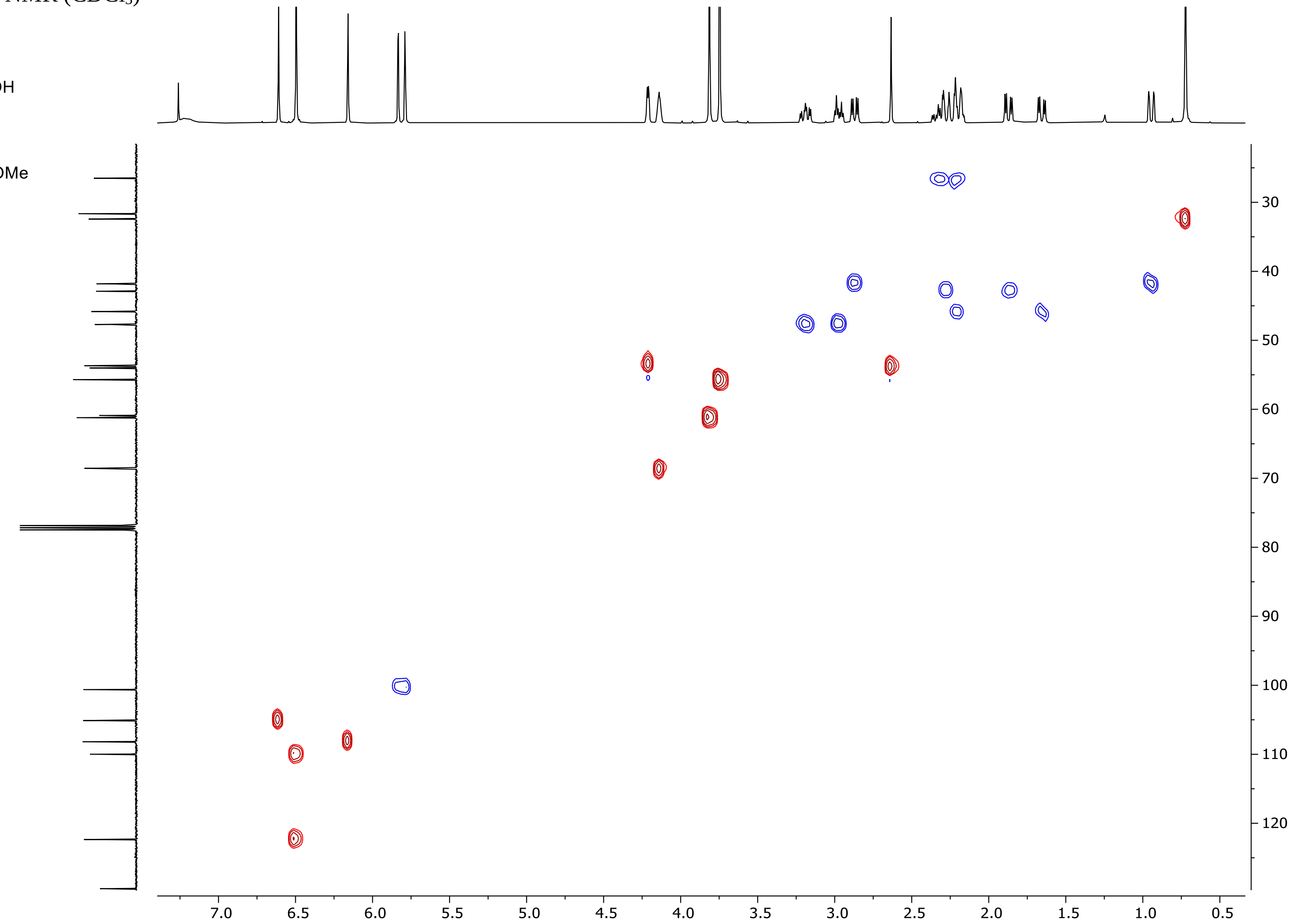

^{1}H–^{13}C HMBC NMR ($CDCl_3$)

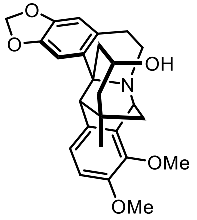

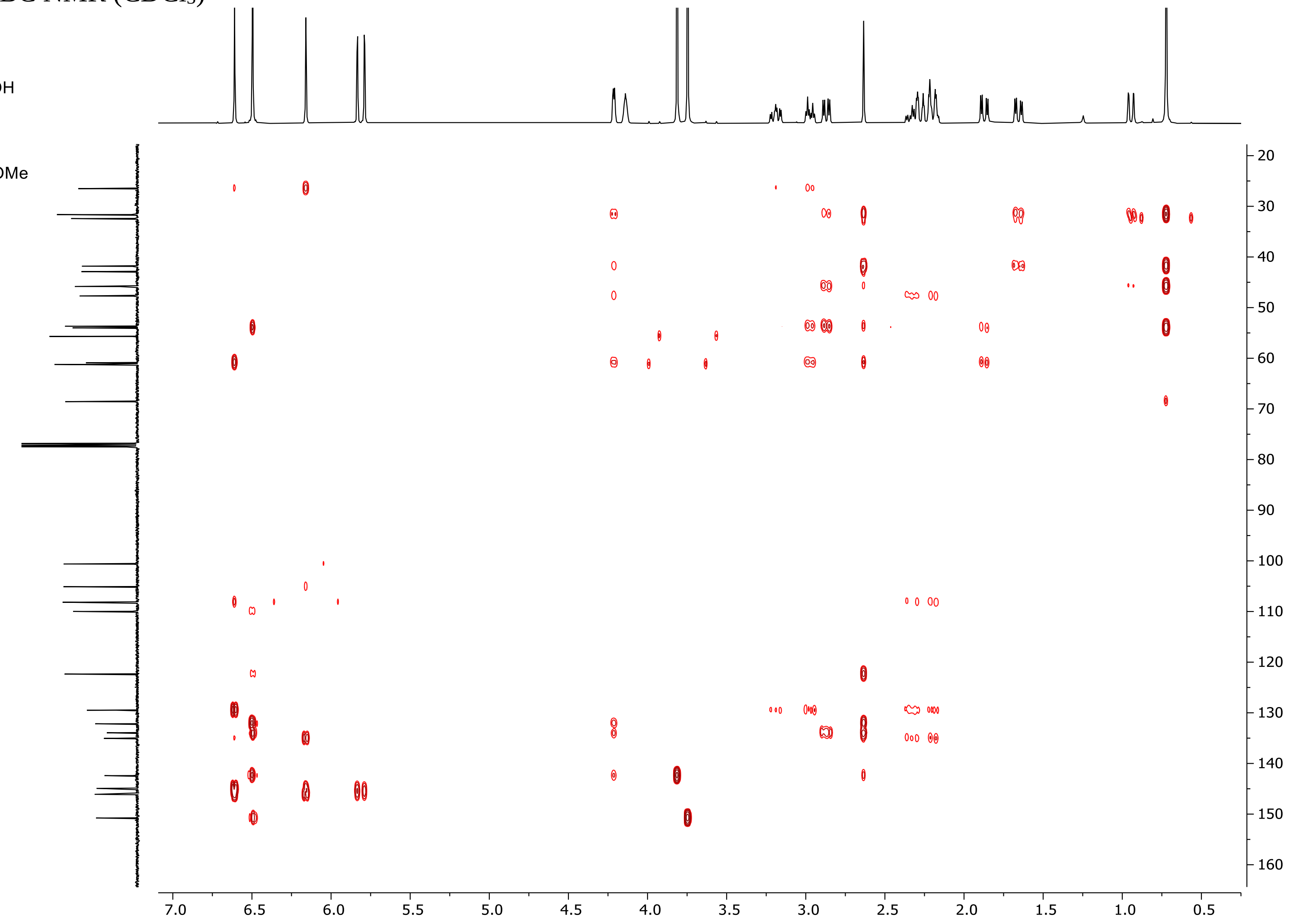

9,10-Dimethoxy-13,15-dimethyl-5,6,8,12b,13,14,15,16-octahydro-8,13-methano[1,3]dioxolo[4',5':6,7]isoquinolino[1,2-*e*]phenanthridin-15-ol (13)

^{1}H NMR (400 MHz, $CDCl_3$)

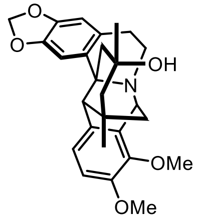

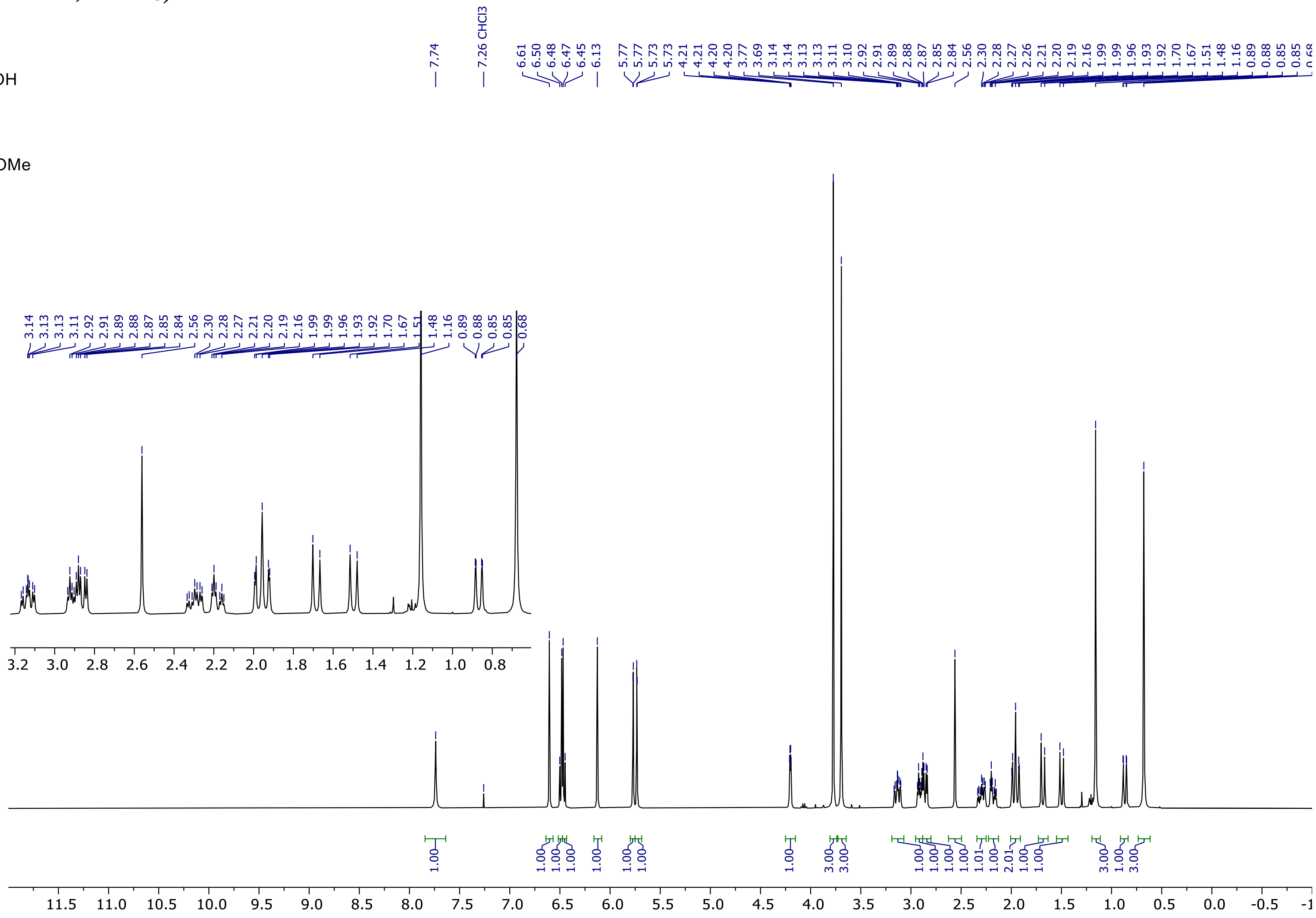

$^{13}C\{^{1}H\}$ NMR (101 MHz, $CDCl_3$)

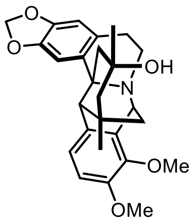

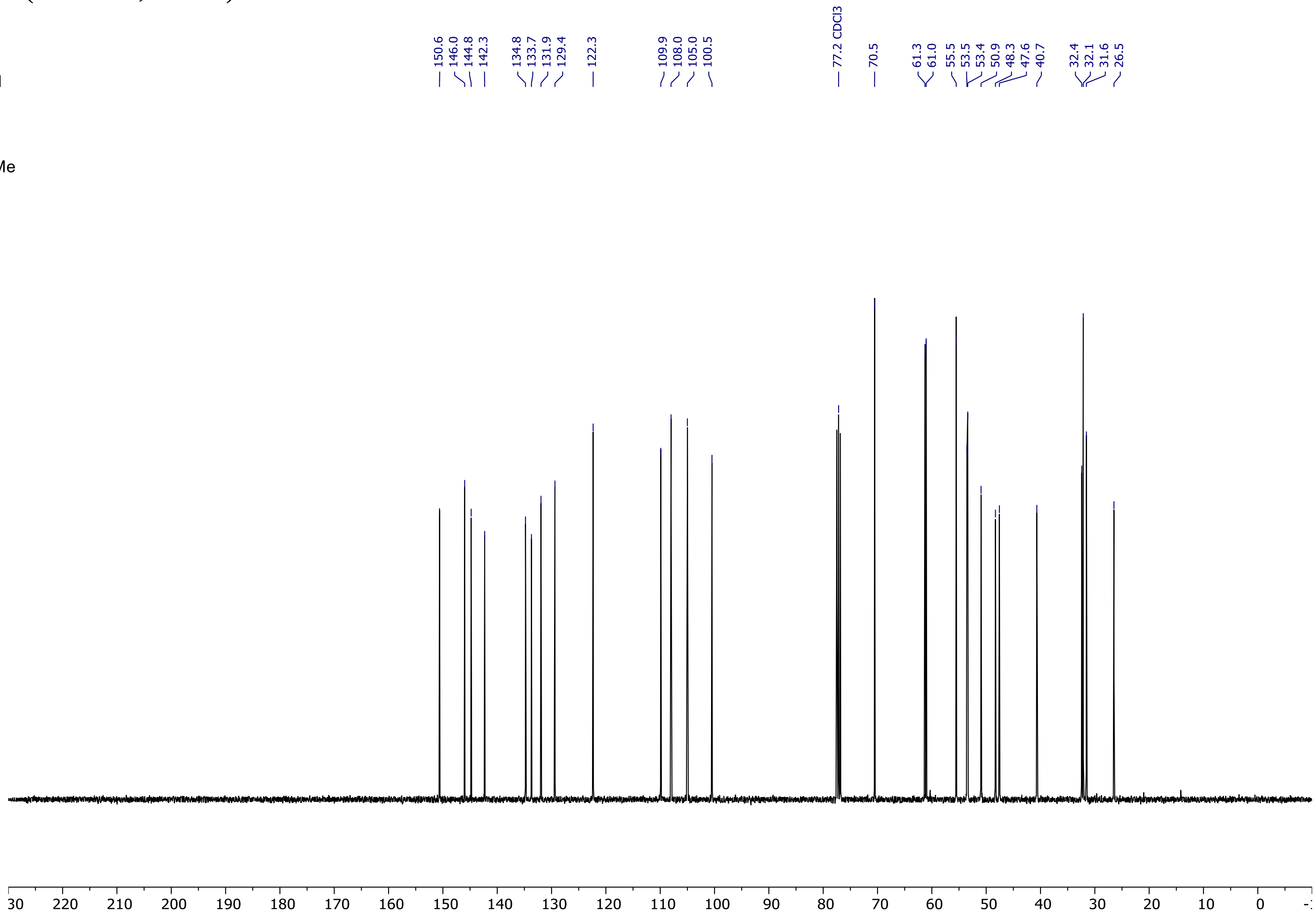

$^{1}H–^{1}H$ COSY NMR ($CDCl_3$)

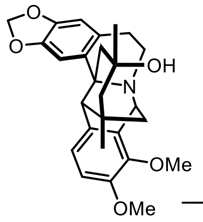

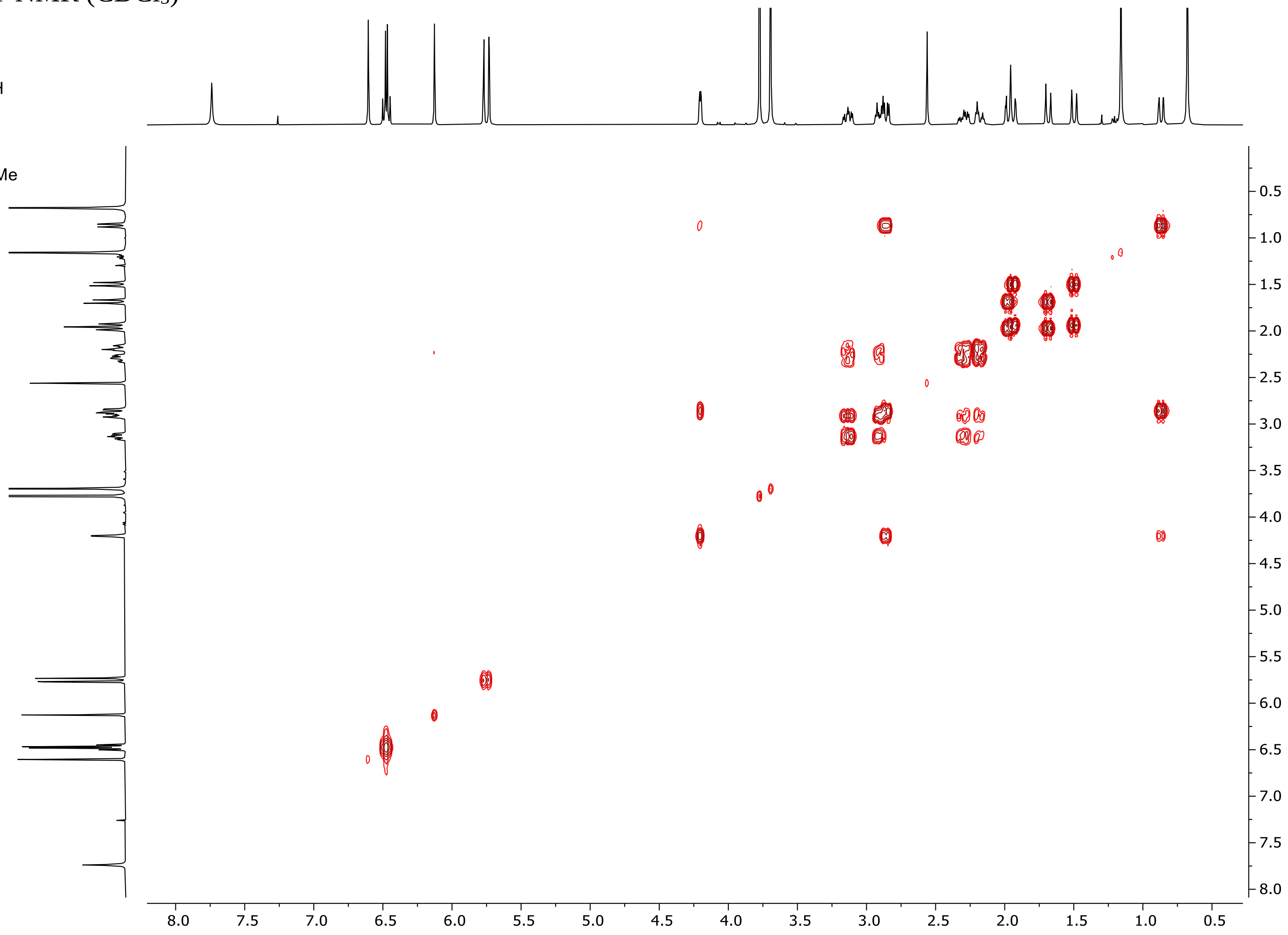

^{1}H–^{13}C HSQC NMR ($CDCl_3$)

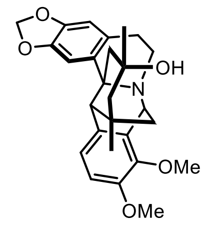

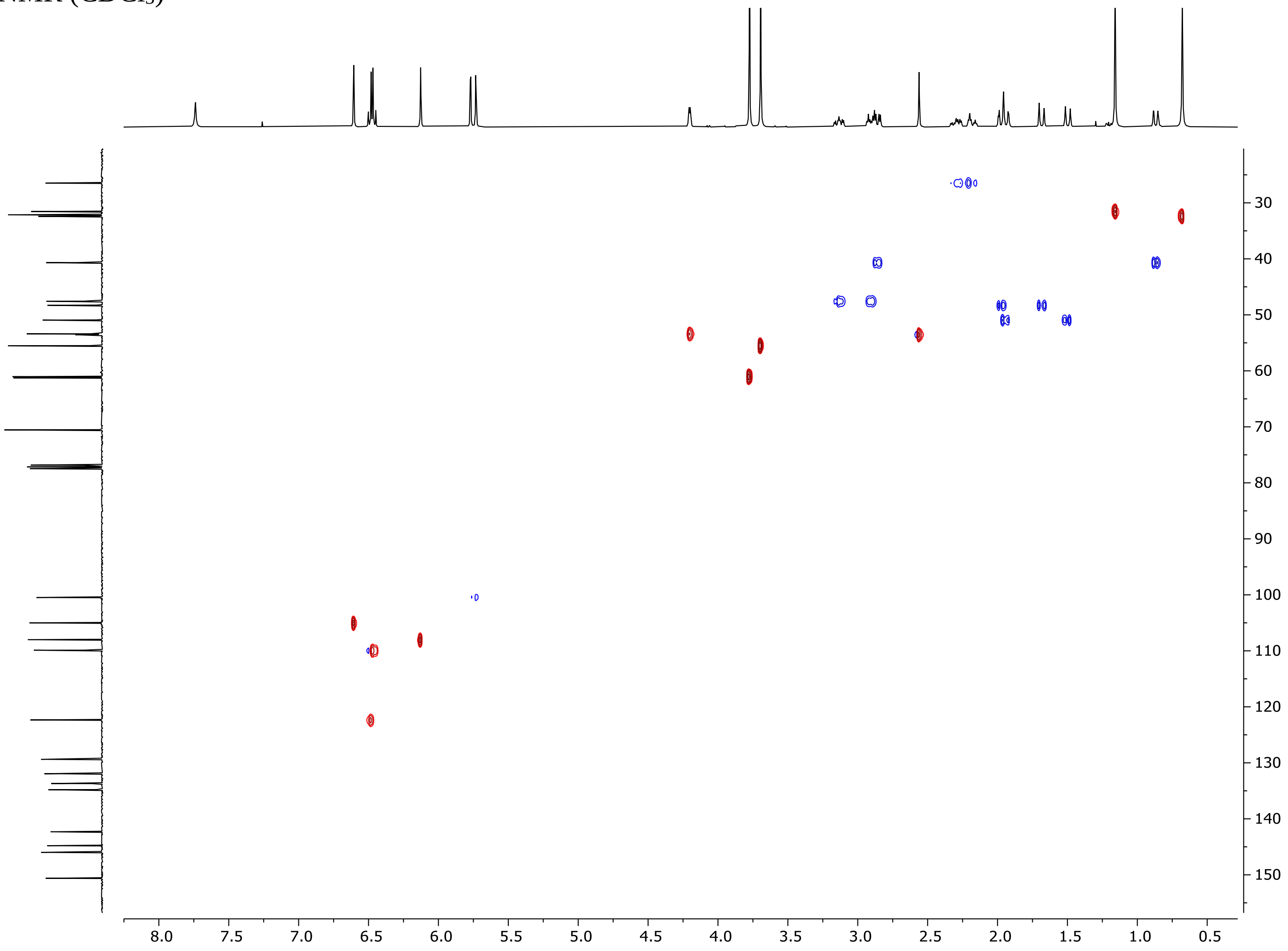

^{1}H–^{13}C HMBC NMR ($CDCl_3$)

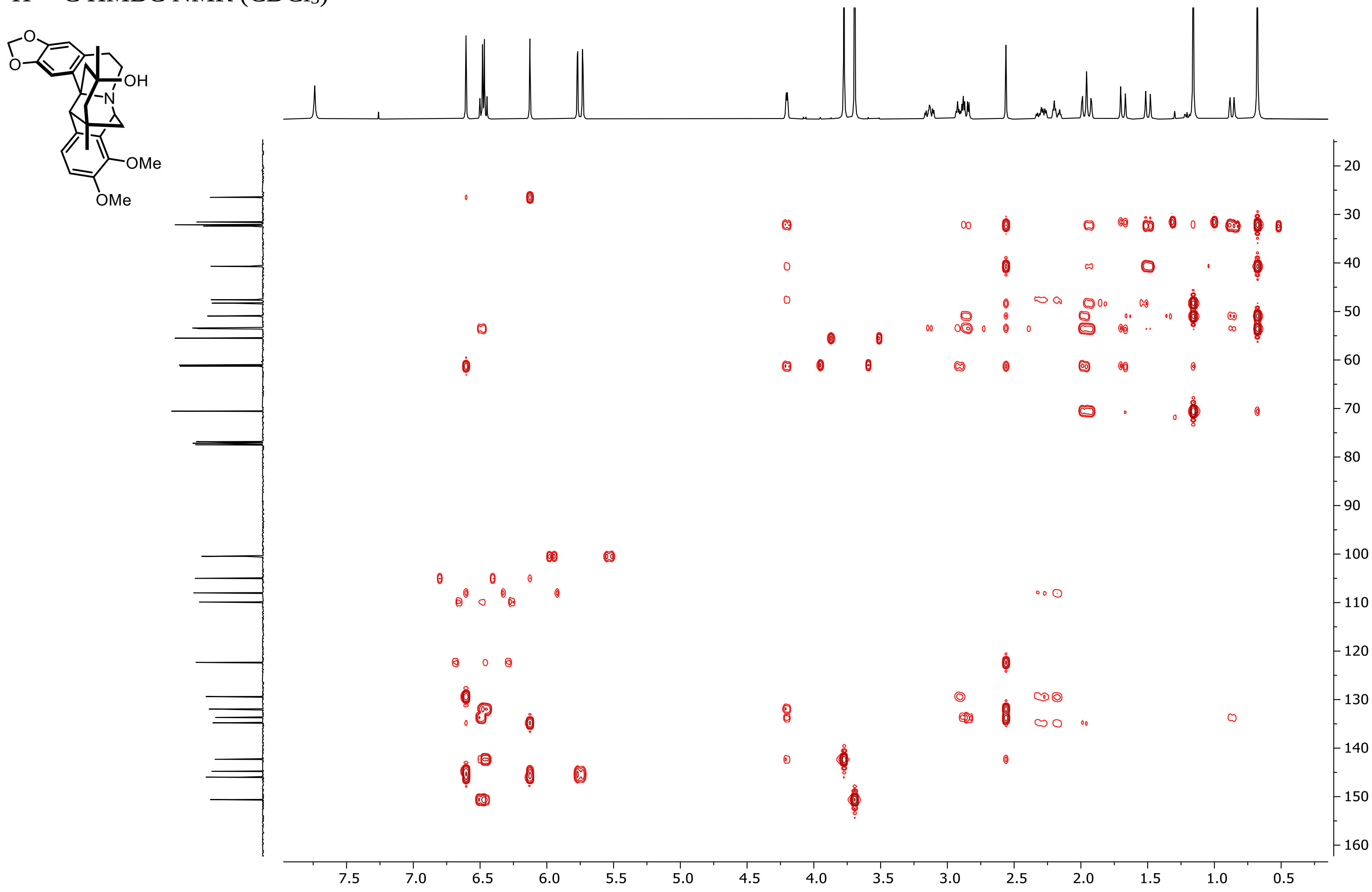

6. References

1. Blasko, G.; Murugesan, N.; Freyer, A. J.; Shamma, M.; Ansari, A. A.; Atta-ur-Rahman. Karachine: An Unusual Protoberberine Alkaloid. *J. Am. Chem. Soc.* **1982**, *104* (7), 2039–2041. DOI: 10.1021/ja00371a049

2. The reference sample was prepared according to the previously published procedure: Lee, G. E.; Lee, H.-S.; Lee, S. D.; Kim, J.-H.; Kim, W.-K.; Kim, Y.-C. Synthesis and Structure–Activity Relationships of Novel, Substituted 5,6-Dihydrodibenzo[*a*,*g*]Quinolizinium $P2X_7$ Antagonists. *Bioorg. Med. Chem. Lett.* **2009**, *19* (3), 954–958. DOI: 10.1016/j.bmcl.2008.11.088

3. Firdous, S.; Freyer, A. J.; Shamma, M.; Rahman, A.-; Urzúa, A. Bridged Protoberberine Alkaloids. *J. Chem. Soc., Chem. Commun.* **1984**, 1371–1373. DOI: 10.1039/C39840001371

4. Shen, X.; Yan, Y.; Li, X.; Ma, J.; Xie, F.; Zhou, S.; Feng, Y.; Yin, T. Isoquinoline Alkaloids from *Thalictrum Glandulosissimum* and Their Network Analysis of Chemotaxonomic Value. *Biochem. Syst. Ecol.* **2022**, *101*, 104390. DOI: 10.1016/j.bse.2022.104390

5. Bi, R.; Yang, X.-N.; Zhou, H.-F.; Peng, L.-Y.; Liu, J.-X.; Zhao, Q.-S. Eleven Undescribed Alkaloids from the Rhizomes of *Sinomenium Acutum* and Their IDO1 and TDO Inhibitory Activities. *Phytochemistry* **2022**, *200*, 113244. DOI: 10.1016/j.phytochem.2022.113244

6. SHELXT: Sheldrick, G.M. SHELXT – Integrated space-group and crystalstructure determination. *Acta Cryst*. **2015**, *A71*, 3–8. DOI: 10.1107/S2053273314026370

7. SHELXL: Sheldrick, G.M. Crystal structure refinement with SHELXL. *Acta Cryst.* **2015,** *C71*, 3–8. DOI: 10.1107/S2053229614024218
